# Feasibility of a dietary life skills course aimed at fostering cooking skills and a healthy diet among university students

**DOI:** 10.1186/s40814-025-01680-y

**Published:** 2025-07-17

**Authors:** Ida Ulrikke Valand, Tormod Bjørkkjær, Charlotte Kiland, Elisabet R. Hillesund, Frøydis N. Vik, Nina C. Øverby

**Affiliations:** 1 Department of Nutrition and Public Health, Faculty of Health and Sport Sciences, University of Agder, Kristiansand, Norway; 2 Department of Political Science and Management, Faculty of Social Sciences, University of Agder, Kristiansand, Norway

**Keywords:** Twenty-first century skills, Food preparation, Cooking classes, Nutrition intervention, Innovative education, Health promotion, Co-creation, Empowerment, Self-determination theory, Young adults

## Abstract

**Background:**

The transition from living at home to independent university life has been associated with deteriorated health outcomes, and many university students lack cooking skills. Life skills education promotes skills essential to mastering everyday challenges such as planning and preparing healthy meals. We developed a dietary life skills course to foster cooking skills and a healthy diet among university students. This paper describes the feasibility testing of this *Skills for Life* course.

**Methods:**

*Skills for Life* was feasibility tested as a pre-post intervention without a control group. The course comprised 10 weekly, practical lessons at a university teaching kitchen. Topics included among others: nutrition from a life course perspective and why diet matters, how to stock your kitchen, how to make the most of your student loan, and sustainable food and ‘food rescuing’. The emphasis was on how to plan and cook easy, cheap, healthy, tasty, and sustainable meals. The students were encouraged to engage with a course-specific website that included short video lectures, relevant literature, learning activities, recipes, and a podcast. An online survey including background information and a validated dietary screener (MyFoodMonth 1.1) was distributed pre- and post-course. Feasibility questionnaires were distributed after each of the 10 lessons and post-course.

Progression criteria were set regarding acceptability (mean value ≥ 4 of 5), demand (30 or more sign up; 20 or more participate), implementation (10 lessons delivered), practicality (intervention delivered within normal working hours) and limited efficacy (no significant adverse effects on participants).

**Results:**

All progression criteria were met. Sixty-nine students signed up, but the dropout rate was substantial. Twenty-eight students attended at least one lesson, and 14 students attended at least five of the 10 lessons. The overall attendance rate was 49%. The course was well received by the students and was assessed as acceptable. Self-perceived course effects such as a healthier diet, increased knowledge of a healthy diet, and improved cooking skills were reported.

**Conclusions:**

The *Skills for Life* course is feasible in a university setting. Further research should investigate how to increase participation to be able to explore possible changes in diet quality in a larger sample.

**Supplementary Information:**

The online version contains supplementary material available at 10.1186/s40814-025-01680-y.

## Key messages regarding feasibility

- University students represent an important group in health promotion. The transition from living at home to independent university life has been associated with adverse health outcomes and deteriorated dietary habits, and many students lack dietary knowledge and cooking skills. Little is known about the feasibility of university courses focusing on diet and cooking among Norwegian university students.
- The key feasibility findings of the present study were that the dietary life skills course was well accepted by the students, but the degree of dropout throughout the semester was high. However, the course was feasible in a university setting, as all the progression criteria were met.
- This feasibility study supports the expansion to a full-scale randomised controlled trial. The following aspects should be addressed: recruit from all levels and not only first-year students, ensure that the course confers credits, reduce the number of lessons or spread them over two semesters, increase the number of participants, and include a control group.

## Background

An important role for universities is to educate and empower students in becoming healthy and literate citizens of the twenty-first century [1–4]. Twenty-first century skills, or life skills, promote literacy and critical thinking [5]. Such skills foster coping in all areas of life [6], and have been associated with improved health [7]. The World Health Organization (WHO) states that a healthy diet is the bedrock for health and well-being [8]. New research has also directed the spotlight toward preconception diets. What young people eat affects not only their current and future health and well-being, but also the health and well-being of their prospective children [9, 10]. In line with the WHO’s life course approach [11], health should be promoted throughout life’s transitions. The act of moving away from one’s parents and starting a life of one’s own is one of these transitions, which are commonly experienced by students entering university. Such transitions are associated with increased sensitivity to both risk and protective factors and represent unique opportunities to improve health and well-being for individuals or generations [11–14].

A recent Norwegian study revealed that university students would benefit nutritionally from increasing their intake of fruits, vegetables, whole grains, and oily fish [15]. Similar findings were reported in a literature review, which revealed that a large proportion of university students have unfavourable eating habits such as low intake of fruits, vegetables, whole grains, fish, and legumes [16]. Furthermore, new research shows that young people today lack dietary knowledge [17]. Qualitative studies have revealed that in their own opinion, many students lack cooking experience [18–21], and that they are interested in enhancing their cooking skills [18]. Cooking and food preparation skills have been associated with healthier eating habits in students [21].

Since poor diet is one of the leading risks for both global and national disease burdens [8, 22], action must be taken to improve it. One way to promote cooking skills and healthy eating in the preconception generation could be to offer a dietary life skills course at universities. Indeed, Wongprawmas and colleagues recognised the dissemination of information about a healthy diet through seminars or courses as one of the key approaches to maintaining healthy eating behaviors when entering university [23]. Previous studies have shown promising results regarding the effects of nutrition-related interventions [24–28]. However, there seems to be a preponderance of theoretical teaching rather than practical cooking in published interventions. We therefore wished to tailor such a course to a Norwegian university setting, with an emphasis on food preparation, to address the lack of cooking experience among students described in the literature. We also believe that students have the right to know that their lifestyle choices affect their potential children’s health. Moderate and varying knowledge of the developmental origins of health and disease (DOHaD knowledge) among young people has been reported previously, alongside a positive association between DOHaD knowledge and dietary outcomes [29, 30]. Therefore, the course included a focus on DOHaD knowledge. We developed *Skills for Life*; a co-created dietary life skills course aimed at new students at the University of Agder (UiA). The course comprised 10 practical cooking lessons and a dedicated website including 10 short video lectures and selected literature and learning activities to complement each lesson.

To evaluate whether an intervention or initiative is perceived as acceptable, in-demand, practical, and effective, a feasibility study is a suitable design [31]. Feasibility studies focus on processes rather than outcomes and are designed to evaluate whether an intervention can work [32]. They are typically conducted as a preparation for a future randomised controlled trial and may be designed in various ways [33]. A well-cited paper on feasibility study design focuses on acceptability, demand, implementation, practicality, adaptation, integration, expansion, and limited efficacy to estimate feasibility [31], which guided the present study. Furthermore, the *Skills for Life* course was developed in close co-creation with the intended users as recommended [34], and was inspired by Self-determination theory (SDT). SDT distinguishes between various types of motivation, presented on a continuum from amotivation through controlled motivation to autonomous motivation [35]. Ryan and Deci explain that it is the quality, rather than the quantity of motivation that predicts behaviour change [35]. Extrinsic motivators such as rewards or punishments have the potential to induce certain behaviours, yet behaviours that are autonomously motivated tend to be more lasting and yield greater impact [36]. The more internalised and self-determined the motivation for a behaviour is, the greater the probability of sustaining the behaviour [36]. Intrinsically motivated behaviours are experienced as meaningful and are performed because of the enjoyment of the activity itself [36]. According to SDT, three psychological needs appear to be fundamental for facilitating intrinsic motivation, learning, development, behaviour change, and well-being [35]. These needs are competence (experiencing accomplishment, knowing what to do and how to do it), relatedness (a feeling of belonging and being supported), and autonomy (lack of external control, making one’s own choices) [37]. Teixeira et al. developed a classification of motivation and behaviour change techniques used in SDT-based health interventions that align with the three SDT needs.

The aim of this study was to evaluate the feasibility of the *Skills for Life* course.

## Methods

### Setting and study design

This study was situated at the UiA in southern Norway. UiA has approximately 14,000 students divided into two campuses and seven academic disciplines (i.e. Business and Law, Engineering and Science, Fine Arts, Health and Sport Sciences, Humanities and Education, Social Sciences, Teacher Education). The *Skills for Life* course was tested during the autumn semester of 2023.

**Fig. 1:**
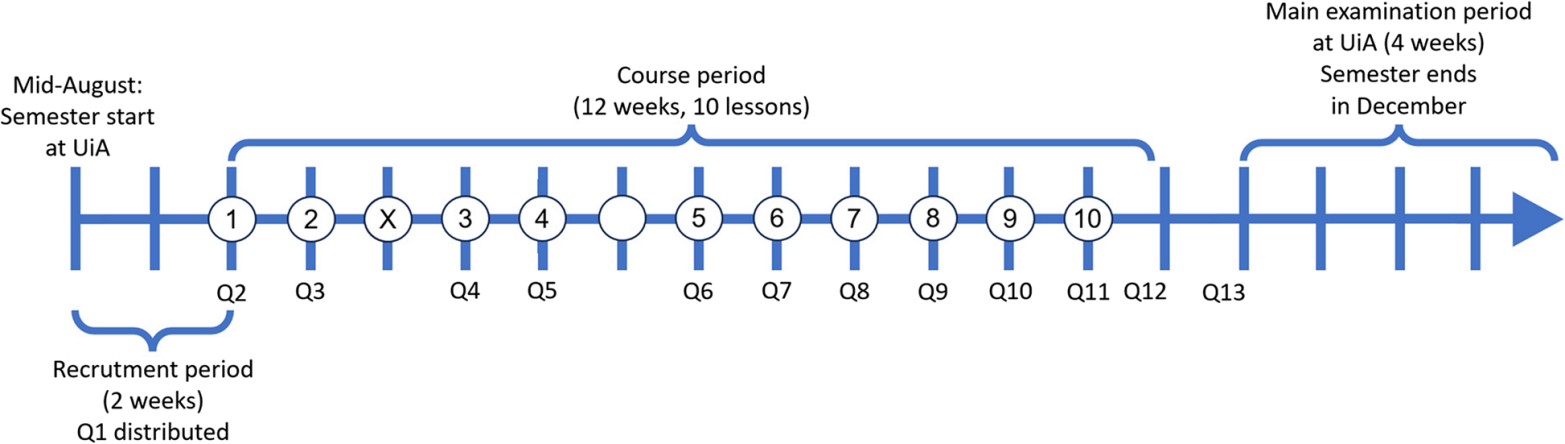
Timeline of the autumn semester of 2023 and the intervention period. White dots with numbers, course lessons at the university teaching kitchen. Two parallel courses/groups per week. White dot with X, lesson cancelled due to illness. White dot, autumn break. UiA: University of Agder. Q1: Questionnaire number 1, etc

The study was conducted as a pre-post intervention without a control group. As we aimed to evaluate feasibility and not primarily course effects, the study was not randomised. The intervention period when participants attended the *Skills for Life* course was 12 weeks (Fig. 1). Recruitment started at the beginning of the semester and lasted for 2 weeks until the course started. The course ended 2 weeks before the main examination period.

### Participants

The course was tailored to first-year students in the transition of moving away from their parents. Approval to recruit through the students’ institutional e-mail was obtained from the university management and a list of 1000 first-year students (500 men and 500 women) was provided. The inclusion criteria when generating the list were as follows: (1) undergraduate student at the campus where the course was delivered and (2) starting in autumn 2023. Students without previous credits were prioritised, and all academic disciplines were represented by both genders. Undergraduate programmes with fewer than 10 students were excluded when generating the list of 1000 students due to the possibility of identifying individuals when reporting the data, to protect the participants’ anonymity.

### Sample size

We aimed to include approximately 30 students in total. This figure was set based on the capacity of the teaching kitchen, which accommodated 16 students. As the teaching kitchen had limited capacity, and to ensure that as many students as possible could find time for the course, we planned for two parallel groups.

#### Recruitment

**Fig. 2:**
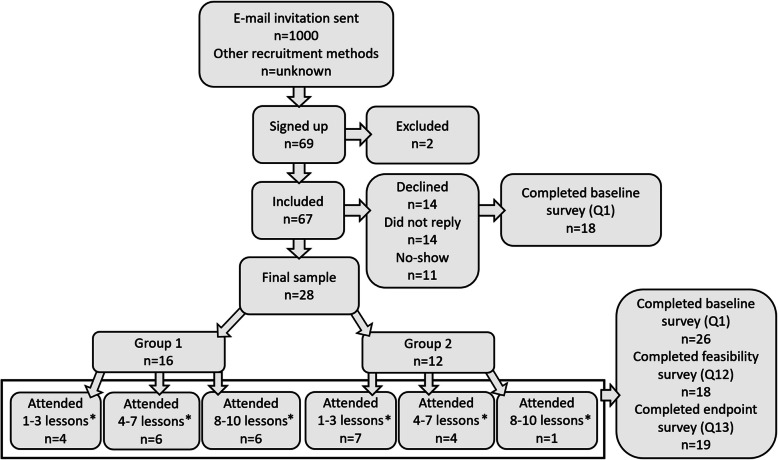
Flow of participants and group attendance. Q1: Questionnaire number 1 etc. * in total

An invitation to participate in the course was sent to the above described 1000 first-year undergraduate students. Other recruitment strategies included social media (university groups on Facebook), videos in communal areas on campus, flyers, a roll-up banner, a stand at the semester start ceremony, and in-person recruitment in classrooms (flow chart in Fig. 2). Relevant information regarding the project and participation was accessible through a study webpage on the university’s website, where students could enrol and provide informed consent to participate. Upon enrolment, participants were asked to choose between two timepoints for when to attend the practical sessions and include information on relevant food restrictions if appropriate.

### Procedures

After the students had registered, the first questionnaire (Q1, Fig. 1) was sent to the 36 first-year students who had registered first. We overbooked to account for potential drop-out, while ensuring that we could manage if all participants attended. The questionnaire and other data collection methods are described below. When the students had completed the baseline questionnaire, we sent them an e-mail regarding where and when the course was delivered, what to bring, and brief information about kitchen hygiene. The e-mail also included a link to the *Skills for Life* website [38] (see screenshots in Additional file 1) and encouragement to watch the first two videos (introduction and lesson 1) as preparation for the first lesson.

After being included, some of the participants reported that they could not attend after all (flow chart in Fig. 2). In that case, new students had the opportunity to attend the course. First-year students were prioritised, but other students were also welcome to fill the remaining places in the course. Students who did not understand Norwegian were excluded, as both the course content and questionnaires were delivered in Norwegian. Some students did not reply or did not show up at the first lesson (*n* = 25). In that case, an e-mail was sent to check that they did not want to attend after all, and new students received the questionnaire and e-mail. In the end, all included students were offered the opportunity to attend the course.

### Questionnaires

The students were asked to complete a total of 13 different surveys, as outlined in Fig. 1 and Table 1. The full questionnaires are provided in Additional file 2. As previously mentioned, the first survey (Q1) was distributed before the course started. This included background information such as age, gender, living conditions, and food preparation habits in addition to two previously validated questionnaires; namely a dietary screener (MyFoodMonth 1.1) and a food literacy questionnaire [39]. MyFoodMonth 1.1 is a rapid dietary assessment tool that was developed by the Lifecourse Nutrition Research Centre and has been validated in first-year students at UiA [40]. Data on food literacy will be presented in a future publication.

**Table 1:** Feasibility measures based on Bowen et al. [31]

Focus area	Instruments	What is measured?	Outcomes	Progression criteria
Acceptability *How does the participants react to the intervention?*	A short survey after each lesson (Q2–Q11) and a survey (Q12) after completion of the entire course	To what extent did the participants - Find the topic important? - Like the course? - Learn something new? - Find the learning activities suitable?	Satisfaction Perceived suitability	3.5 of 5 or more on each parameter Mean value of 4.0 or more on all parameters
Demand *Estimated or documented use of intervention activities*	Course instructor’s notes A survey (Q12) after completion of the entire course	How many signed up and attended? How much did they use the website, videos, recipes, podcasts, etc.?	Actual use Perceived demand	30 or more sign up 20 or more participate
Implementation *Was the intervention implemented as planned and proposed?*	Course instructor’s notes A survey (Q12) after completion of the entire course	Experiences with recruitment and execution of the course	Degree of execution	10 lessons delivered
Practicality *To what extent was the intervention implemented in this setting?*	Course instructor’s notes	How much time and money were spent?	Type and amount of resources needed	Intervention delivered within normal working hours for course instructor and assistants
Limited efficacy *Testing the intervention with limited statistical power (small sample, no control group)*	Survey including background information and dietary screener before and after the course (Q1 and Q13) Course instructor’s notes	Possible changes in diet quality score and dietary habits after completion of the course Potential adverse effects were tracked	Intended effects Effect-size estimation	No significant adverse effects observed or reported from participants

*Q1* questionnaire number 1 etc

### Assessment of diet quality and food preparation habits (limited efficacy)

The dietary screener was used to assess how often the participants consumed 33 different food groups in the previous month to evaluate alignment with the then-current Norwegian dietary guidelines [41] before and after the *Skills for Life* course. The response options for the frequency of intake originally included: never, once a month, 2–3 times a month, once a week, 2–4 times a week, 5–6 times a week, once a day, 2–3 times a day, 4–5 times a day, and 6 or more times a day [40]. The options ‘once a month’ and ‘2–3 times a month’ were excluded by mistake when the questionnaire was generated. Thus, the category ‘never’ was changed to ‘rarely/never’.

Food preparation habits were measured via a previously used [42] question about the frequency of cutting fruit and vegetables and cooking dinner from scratch.

### Assessment of acceptability, demand and implementation

A short online survey (Q2-11, Table 6) was sent during or after each lesson to the participants attending the respective lesson. When there was time, the participants were encouraged to complete the survey at the end of the session before leaving the kitchen. The students were asked to evaluate the lesson using a 5-point scale represented by emotion icons ranging from angry to sad, neutral, happy, and smiley faces, according to their degree of agreement or disagreement with six statements (Table 6). Using emoji response categories has previously been found to be a suitable tool for data collection among undergraduate students [43]. In the present study, this was chosen as a quick and easy method to reduce the pressure on participants. In addition to the evaluation statements, an optional comment field was included in case they had additional feedback. The acceptability statements in Q2-12 were inspired by a school-based feasibility study on reducing sugar-sweetened beverage consumption [44].

A few days after the completion of the 10 cooking lessons, a similar but somewhat extended survey (Q12, Table 7) regarding the entire course was sent to the 28 participants who had attended at least one lesson. In addition to the acceptability statements, this survey included questions regarding the reason for signing up, how they learned about the course, motivators and barriers for attending, whether they noticed any course effects in everyday life, and the use of the online resources. Up to two reminders were sent, if necessary, to increase the response rate of the surveys (Q1-12).

One week after distributing the feasibility survey regarding the entire course (Q12), the post-course questionnaire (Q13) was distributed to the 28 course participants to explore potential differences in diet from baseline to endpoint. To increase the response rate, a NOK 200 gift card for the student canteen was offered to all those who completed the survey. A reminder was sent to those who had not completed the post-course questionnaire after 2 weeks.

### Outcomes

To assess the feasibility of the *Skills for Life* course, we focused on the following aspects mentioned by Bowen et al. [31]: acceptability, demand, implementation, practicality, adaptation, and limited efficacy. The other Bowen aspects adaptation, integration, and expansion were excluded because they were judged as falling outside the scope of the project. The course instructor systematically recorded attendance, estimated time consumption, costs, and potential adverse effects for each lesson. In this study, participation was defined as attending a minimum of one cooking lesson. Progression criteria were set in conjunction with data reporting, and not a priori as recommended [45]. Bowen and colleagues [31] provided no explicit guidelines regarding such criteria. Thus, we employed our best judgement to define cutoffs that seemed appropriate and compliant with high-quality procedural standards. An overview of the feasibility measures and progression criteria is presented in Table 1.

### The *Skills for Life *course

The aim of the *Skills for Life* course was to improve students’ cooking skills and promote a healthy diet in line with the then-current Norwegian dietary guidelines [41]. The course development was initially guided by 13 focus group discussions with 57 UiA students from all seven disciplines [18] and literature reviews. The main findings from the focus group discussions were that the students were interested in learning about diet and health, especially how to plan and cook quick, easy, healthy, tasty, affordable, and sustainable meals. Personal benefits were highlighted, and they wanted the course to be practical, interactive, well-composed, and at a suitable level. Cooking lessons and having dinner at the course were motivators. The perceived barriers were lack of relevance, appeal, or if the course was too demanding, demotivating, or expensive [18]. These findings were accounted for when developing the course by emphasising the interactive cooking lessons, including a communal meal, supplemented by videos and additional learning materials accessible through the course-specific website. Furthermore, participation in course sessions and completion of homework were voluntary, and the course was offered at no cost to the participants. Preliminary course content was discussed in three new focus groups, involving 12 of the 57 students. Selected learning activities were tested and evaluated by undergraduate students in public health nutrition. Scripts for the short video lectures were read and evaluated by a reference group comprising six undergraduate students (two men and four women) from different academic disciplines. These data were not published, but the main finding was that the preliminary course content was well received. In response to feedback from the students, the course content was further refined and developed. The co-creation of the course was led by the first author and included inputs from the research team and two research assistants from the Lifecourse Nutrition Research Centre at UiA. The course did not confer credits, but students who met the attendance rate of 80% were awarded a course certificate.

The *Skills for Life* course was inspired by SDT, emphasising the support of students’ needs for competence, relatedness, and autonomy [35]. It began with simple recipes to establish foundational skills and progressively increased the complexity of recipes and assignments as the course advanced. For example, recipes were provided for the first three lessons, but in the fourth lesson, the participants were encouraged to use their creativity and cook without a recipe. The students worked in groups so that they could help each other, and the course instructor and assistants were available for questions and provided guidance during the lesson. Furthermore, students’ need for relatedness was supported by the sociable nature of the course. They cooked and ate together, and everybody joined in the communal meal at the end of each session. Compared with conducting an online course, choosing to include practical cooking lessons in the teaching kitchen was important to promote competence and relatedness. In line with the motivation and behavior change techniques suggested by Teixeira et al. [46], competence was supported by (1) offering constructive feedback during the lessons regarding group assignments, cooking techniques, and cooked dishes, (2) helping to develop a clear and concrete action plan by suggesting tools such as the plate model to plan and cook healthy meals, and (3) for those who engaged with the videos and other learning activities at the website, setting a goal regarding dietary advice they wanted to work on improving. Moreover, the following relatedness-support techniques were applied, corresponding to Teixeira and collaborators’ suggestions [46]: (1) encouraging the participants to ask questions, (2) expressing positive support regardless of their success or failure, (3) showing interest in each participant, e.g. by learning their names and asking about their experiences regarding diet and cooking as well as more personal aspects when appropriate, and (4) using empathic listening, e.g. during the meal situation, and by asking for permission to provide guidance, for example regarding cooking techniques. Finally, the students’ need for autonomy was supported by the open and flexible nature of the course. The course and lessons were voluntary, and they could choose who to work with, what to cook, and what to eat. Furthermore, the students could engage with website activities at their own discretion. Corresponding to Teixeira and colleagues’ suggestions [46], the following autonomy-support techniques were used: (1) using informational, non-judgemental and non-controlling language, (2) providing a meaningful rationale for behaviour change by highlighting motives for eating in line with the dietary guidelines, (3) providing choices, and (4) for those who engaged with the website, encouraging reflection and exploration of participants’ own behaviour to inform personal choices.

The co-created dietary life skills course comprised 10 lessons with the following topics:

1. Nutrition from a life course perspective and why diet matters
2. How to eat healthily
3. How to store food to avoid food waste
4. How to stock your kitchen
5. Preconception diet
6. Food labels and how to interpret them
7. How to make the most of your student loan
8. Sustainable food and “food rescuing”
9. What is true about food and health?
10. Cooking competition and mindful eating

The participants were invited to attend 10 weekly cooking lessons at the teaching kitchen on campus (timeline in Fig. 1). Each lesson lasted approximately 2.5 h. Here, they engaged in discussions and group work, including meal planning, food preparation, and cooking.

The first author was experienced in teaching practical cooking and delivered the course. In most of the lessons, two undergraduate students in public health nutrition (internship) were observers, helped with practical issues, and were available to answer questions from the participants. When the internship students were unavailable, one or two of the course instructor’s colleagues in practical cooking stepped in to fill this role (with the exception of two lessons that were led by the course instructor alone).

Each lesson started with information regarding the current topic, including brief theory and a summary of the corresponding video lecture. This short introduction was followed by a demonstration or assignment, mostly in groups. After this, the students cooked in pairs or groups and set the table for a communal meal. The necessary foodstuffs were available, including suitable substitutes in case of food allergies or other dietary restrictions. The students were encouraged to consider both their own dietary needs and, if feasible, those of others, ensuring that everybody could taste the various dishes. During the meal, the participants, instructor, and observers shared the dishes, while engaging in conversations about the current topic and everyday discussions. The dialogue also included evaluations of the dishes and cooking processes, as well as exchanges of experiences related to food and meals. After the meal and a summary of learning outcomes, everybody participated in cleaning the kitchen, and the cooked leftovers were shared between the participants to take home. A more detailed overview of the course and lessons can be found in Additional file 3.

To gain a deeper understanding of the more theoretical aspects of planning and managing a healthy diet, the participants also gained access to the *Skills for Life* website, which comprised a short video lecture (3–12 min) and selected literature and learning activities for each lesson. A podcast of seven episodes and 35 recipes was also included on the website. The website, video lectures, and learning activities were developed by the research team, assisted by the IT and Communication departments at UiA. The podcast was produced by the first author, and different UiA colleagues and two first-year students were interviewed. The recipes were developed by the Lifecourse Nutrition Research Centre. All the food groups and various meal types including breakfast, lunch, dinner, and healthier desserts/snacks were represented among the recipes, with an emphasis on quick, easy, healthy, cheap, tasty, and sustainable dinners for students. The food choices for both the recipes and the course in general were based on the then-current Norwegian dietary guidelines [41], with a particular focus on vegetables, fish, legumes, and whole grains. These foods can be both easy and quick to cook, and they represent a healthy, cheap, and sustainable option for meat [47].

In accordance with the flipped classroom approach [48], the students were encouraged to watch the corresponding video before attending the practical cooking lesson, and to work with the corresponding literature and learning activities after each lesson. The first two online lessons, the podcast, and the recipes were available at the *Skills for Life* website [38] when the course started. After this, the eight remaining online lessons were published on the website weekly.

### Statistical analyses

The data were processed and analysed via IBM SPSS Statistics version 29 and Microsoft Office Excel. Descriptive statistics, i.e. frequencies and percentages, were used to present the sample characteristics. It should be noted that, owing to rounding, percentages may not necessarily total 100%. Normality was assessed via the Shapiro–Wilk test, which is suitable for small sample sizes (*n* < 50) [49]. Data are presented as median values with interquartile ranges for skewed data and means with standard deviations for normal data. Means and medians with corresponding measures of dispersion were calculated using descriptive statistics and frequency analysis. Participants in the two different groups (groups 1 and 2, see flow chart in Fig. 2) were pooled when analysing and presenting data. Owing to the small sample size and lack of statistical power, comparative tests of potential differences in pre- and post-data were not appropriate.

The background question regarding whether the participants could afford healthy food (Table 3) originally utilised a five-point Likert scale. However, the data were pooled into three categories for analysis and presentation.

To calculate the diet quality score (Table 8), the variables were recoded to have the same denominator, i.e. times per day. Food groups that were reportedly consumed never/rarely received a value of 0. If a food group was reported to be consumed 2–4 times a week, the middle value (3) was divided by the number of days in a week (3/7 = 0.43). A value of 6 was given for food groups that were reportedly consumed 6 or more times a day. The variables were then pooled into 10 diet components as suggested by Salvesen and colleagues [40]. Based on alignment with the dietary guidelines, each component had either positive or negative scoring, ranging from 0 to 10. We used the same scoring as Salvesen et al., except that we changed the score for eating fish once a week from 10 to 8.5. This corresponds better with the guidelines that recommended eating fish 2–3 times weekly [41]. A detailed overview of the diet components and scoring system is available in Additional file 4. The diet component scores were combined into a total diet quality score ranging from 0 to 100. A high score indicates high diet quality in line with the dietary guidelines. The values regarding food preparation habits (Table 9) were recoded as follows: never = 0, less than once a week = 0.1, once a week = 1, twice a week = 2 and so on, until every day (or more) = 7.

To calculate the acceptability scores, the data from each of the 10 lessons were merged. Each participant’s score for each question was summarised, and the mean value was calculated by dividing the total score by the corresponding number of responses from the participant.

## Results

### Implementation

The intervention was implemented as planned and proposed. In accordance with the progression criteria, 10 lessons were delivered. Among the 16 students (57%) who reported the recruitment strategy through which they initially became aware of the intervention, 7 (44%) reported flyers, 5 (31%) reported roll-up, and 5 (31%) reported that they had received e-mail invitations (Table 2, multiple answers were possible). None reported learning about the course through social media.

**Table 2:** Number of participants recruited through different strategies, measured post-intervention (*n* = 18)

How did you find out about the course?	*n*^a^ (%)
Flyers	7 (44%)
Roll-up banner	5 (31%)
E-mail invitation	5 (31%)
Videos on campus	3 (19%)
Semester start ceremony	3 (19%)
In-person recruitment	3 (19%)
Friend/peer	1 (6%)
Social media	0 (0%)
Do not remember	2 (13%)

^a^Two missing. Percentages are calculated based on the number of responses for this question. Multiple answers were possible

### Demographics and demand

A total of 69 students signed up, but many dropped out due to time constraints (Fig. 2). Twenty-eight participants attended the course at least once; however, one of them did not complete any of the questionnaires. In addition to 26 participants, 18 students completed the baseline survey (Q1) but were unable to attend the course. This resulted in a total of 44 responses for the baseline survey.

Baseline data for both course participants and non-attenders are detailed in Table 3.

**Table 3:** Baseline data for course participants and non-attenders

	Attended course (*n* = 28^a^)	Did not attend course (*n* = 18)
Men, *n*	10 (36%)	7 (39%)
Women, *n*	18 (64%)	11 (61%)
Age (years), median (IQR)	20.0 (19.0–21.0)	20.0 (19.7–22.2)
Living conditions, *n* - Alone - Shared housing - With parents/family member(s) - With partner	5 (19%) 16 (62%) 3 (12%) 2 (8%)	4 (22%) 10 (56%) 2 (11%) 2 (11%)
Main responsibility for cooking, *n* - Yes - No - Shared responsibility	16 (62%) 2 (8%) 8 (31%)	11 (61%) 3 (17%) 4 (22%)
Responsible for children, n	0 (0%)	0 (0%)
Kitchen/access to a kitchen is limiting for diet/cooking, *n*		
- A lot - Somewhat - Not at all	5 (19%) 9 (35%) 12 (46%)	3 (17%) 10 (56%) 5 (28%)
Income beyond student grants/loans, *n*	10 (38%)	8 (44%)
Can afford healthy food, *n* - Strongly disagree/disagree - Neutral - Agree/strongly agree	4 (15%) 8 (31%) 14 (54%)	7 (39%) 4 (22%) 7 (39%)
Cutting vegetables (times a week (t/w)), median (IQR)	3.0 (2.0–4.2)	2.0 (0.8–4.2)
Cooking dinner from scratch (t/w), median (IQR)	3.5 (1.7–6.0)	2.0 (0.8–5.0)
Cutting fruit (t/w), median (IQR)	1.5 (0.1–3.0)	1.0 (0.1–3.0)
Total diet quality score, mean (SD)	49.5 (17.2)	42.1 (18.5)

^a^2 missing, except for gender. Percentages are calculated based on the number of responses for each question

Percentages may not add up to 100% due to rounding

*SD* standard deviation, *IQR* interquartile range

Ten men (36%) and 18 women (64%) participated in *Skills for Life* (Table 3). The 26 participants who completed the baseline survey were between 18 and 35 years old (median age 20.0 years (IQR 19.0–21.0)). The majority (*n* = 16, 62%) lived in shared housing, and none were responsible for children. Although only 10 students (38%) reported having an income beyond student grants and loans, most of them (*n* = 14, 54%) agreed or strongly agreed that they could afford healthy food. The participants reported cutting vegetables 3.0 times a week, cooking dinner from scratch 3.5 times a week, and cutting fruit 1.5 times a week at baseline. Their mean diet quality score was 49.5 out of 100 at baseline (Table 3).

Most of the participants were first-year students (89%), but three were third-year students (11%). Five participants (18%) had studied something else before, even though they reported being first-year (*n* = 4) or third-year (*n* = 1) students. All seven academic disciplines at the university were represented (Table 4).

**Table 4:** Participants’ academic disciplines and study programmes (*n* = 28)

Academic discipline	Study programmes	n (%^a^)
Social Sciences	Political Science Global Development Studies Sociology	12 (43%)
Business and Law	Economics and Administration Law Marketing and Management	5 (18%)
Health and Sport Sciences	Nursing	4 (14%)
Humanities and Education	English History Communication	3 (11%)
Engineering and Science	Mathematics	2 (7%)
Fine Arts	Music	1 (4%)
Teacher Education	Teacher, level 5–10	1 (4%)

^a^ Percentages may not add up to 100% due to rounding

### Attendance

Between 9 and 20 students attended each lesson (Table 5). The overall attendance rate was 49%. Eleven students (39%) attended 1–3 lessons, 10 students (36%) attended 4–7 lessons, and seven students (25%) attended 8–10 lessons (Fig. 2). Three men did not enrol until prior to the 2nd lesson, whereas one woman signed up the day before the 3rd lesson.

**Table 5:** Attendance (demand). Total number of participants varied across the first three sessions. Maximum enrolment was 28 participants

Lesson	Topic	Number of participants	Attendance rate
1	Nutrition from a life course perspective and why diet matters	20 out of 24 (14 women, 70%)	83%^a^
2	How to eat healthily	20 out of 27 (14 women, 70%)	74%^a^
3	How to store food to avoid food waste	20 out of 28 (13 women, 65%)	71%
4	How to stock your kitchen	17 out of 28 (13 women, 76%)	61%
5	Preconception diet	10 out of 28 (8 women, 80%)	36%
6	Food labels and how to interpret them	9 out of 28 (6 women, 67%)	32%
7	How to make the most of the student loan	10 out of 28 (7 women, 70%)	36%
8	Sustainable food and “food rescuing”	9 out of 28 (5 women, 56%)	32%
9	What is true about food and health?	9 out of 28 (7 women, 78%)	32%
10	Cooking competition and mindful eating	12 out of 28 (8 women, 67%)	43%

^a^ Three students did not enrol until the 2nd lesson, and one student enrolled prior to the 3rd lesson

### Usage of online resources

Most of the 18 participants who completed the post-course feasibility questionnaire (Q12) reported using the website and watching videos. Four out of the 18 students (22%) used the website weekly, and eight participants (44%) used it 2–3 times a month. Three students (17%) used it once a month or less, whereas three students (17%) never visited the website. Twelve participants (67%) watched some of the 11 videos (1–6 videos), whereas only three (17%) watched 7 or more. Three students (17%) watched none of the videos. Six participants (33%) worked with the learning activities online, and only three participants (17%) checked the literature. None of the participants listened to the podcast episodes. Eight of the students (44%) used some of the recipes at home.

### Practicality

In line with the progression criteria, the course was delivered within normal working hours for both the course instructor and the assistants. Each lesson lasted between 2 h and 15 min and 3 h; typically averaging approximately 2.5 h. Practical preparations varied according to the topic and ranged from 10 min (for only the course leader) to 3.5 h (total time spent by the course leader and two observers), with most requiring 1 h or less. The average preparation time was 1.5 h. Practical preparations included presenting foodstuffs and printing recipes and assignments for all lessons. For certain lessons, dishes such as slow-risen bread and slow-cooked meat required advance preparation because of their extended preparation time. In lesson 7, we simulated a food store by attaching price tags to all food items used, enabling students to calculate the cost of their dishes. These specific activities necessitated longer preparation times than the average lesson did.

The food was ordered through the university’s standard system. The time spent on lesson planning, food ordering, preparing PowerPoint presentations, and other related tasks was additional to practical preparations and was not estimated in this project. Practical work after each session typically lasted 30–60 min, and the mean daily cost for food items was NOK 700. This resulted in a per-person cost of NOK 71.0, which was calculated by summing the attendance of each participant, the course instructor, and observers. Other expenses, such as energy costs for preparing the food, maintenance of kitchen facilities, and instructor wages, were not calculated in this project.

### Acceptability

The acceptability outcomes are detailed in Tables 6 and 7. The students found the lessons and the entire course to be important, likable, and acceptable. They also thought that the lessons and course would be important for other students and that the group work, activities, and cooking were appropriate, educational, and useful. All the statements received high median ratings, ranging from 4.5 to 5 out of 5, well above the progression criteria of at least 3.5 out of 5 for each parameter and a mean value of 4.0 or more for all the parameters.

**Table 6:** Acceptability results from short surveys after each lesson (*n* = 27)

Statement	Median^a^ (IQR)
The topic of this lesson is important for me to learn about	4.80 (4.50–5.00)
I liked this lesson	5.00 (4.90–5.00)
I learned something new from this lesson	4.83 (4.33–5.00)
Other students would like this lesson	5.00 (4.80–5.00)
It is important for students to attend this lesson	4.67 (4.50–5.00)
The group work, activities and cooking were appropriate, educational, and useful	5.00 (4.67–5.00)

^a^Average across 10 lessons. 5-point scale represented by emotion icons from 1  to 5  . Of 136 possible responses, 9 are missing. Surveys were sent only to those attending each lesson

*SD* standard deviation, *IQR* interquartile range

**Table 7:** Acceptability results from a short survey after the entire course (*n* = 18)

Statement	Median^a^ (IQR)
The topic of the Skills for Life course is important for me to learn about	5.00 (4.75–5.00)
I liked the Skills for Life course	5.00 (5.00–5.00)
I learned something new from the Skills for Life course	5.00 (5.00–5.00)
Other students would like the Skills for Life course	5.00 (4.75–5.00)
It is important for students to attend the Skills for Life course	4.50 (4.00–5.00)
The group work, activities, and cooking in the practical cooking sessions were appropriate, educational, and useful	5.00 (4.00–5.00)

^a^ 5-point scale represented by emotion icons from 1  to 5  . The survey was sent to all participants who had attended the course at least once. Ten of the 28 participants did not respond

*SD* standard deviation, *IQR* interquartile range

### Limited efficacy

Pre- and post-data could be successfully matched for 19 participants. As detailed in Table 8, the students had a moderate diet quality both before (47.2 out of 100) and after (50.3 out of 100) the course. The median legume score increased from 0.0 to 4.0 post-course.

**Table 8:** Diet quality score before and after completion of the course (*n* = 19)

	Before course	After course
	Median (IQR)	Median (IQR)
Whole grains	10.0 (4.0–10.0)	10.0 (8.0–10.0)
Vegetables	6.0 (4.0–9.0)	6.0 (4.0–8.0)
Legumes	0.0 (0.0–4.0)	4.0 (0.0–4.0)
Fish	10.0 (8.5–10.0)	10.0 (0.0–10.0)
Meat	4.0 (2.0–4.0)	4.0 (2.0–4.0)
Unsalted nuts and seeds	0.0 (0.0–0.0)	0.0 (0.0–4.0)
Sugar-sweetened beverages	6.0 (1.0–10.0)	4.0 (1.0–10.0)
Sugary foods	4.0 (0.0–4.0)	4.0 (1.0–4.0)
Salty foods	6.0 (4.0–10.0)	6.0 (6.0–10.0)
	Mean (SD)	Mean (SD)
Fruits and berries	4.2 (3.1)	4.2 (2.8)
Total diet score	47.2 (18.9)	50.3 (12.6)

Data from those who attended the course at least once and completed both Q1 and Q13. Nine of the 28 participants did not respond

*SD* standard deviation, *IQR* interquartile range

Table 9 details the frequency of food preparation habits before and after the course. The frequency of cutting vegetables and cooking dinner from scratch remained similar after completion of the course; however, an increase in the frequency of cutting fruit could be discerned. Owing to a lack of statistical power, this cannot be confirmed by statistical analyses.

**Table 9:** Frequency of food preparation habits (times per week) pre- and post-course (*n* = 19)

	Before course	After course
	Median (IQR)	Median (IQR)
Cutting vegetables	3.6 (1.9)	3.5 (2.1)
Cooking dinner from scratch	3.9 (2.3)	3.7 (2.3)
	Mean (SD)	Mean (SD)
Cutting fruit	1.6 (1.8)	2.7 (2.5)

*Data from those who attended the course at least once and completed both Q1 and Q13. Nine of the 28 participants did not respond*

*SD* standard deviation, *IQR* interquartile range

During the ninth session, immediately before the meal, one student experienced illness and seemed to be on the verge of fainting. However, she rapidly regained her composure after lying down briefly and subsequently consuming food and drink. Considering that the student returned for the final session, we evaluated this incident as a minor adverse event possibly attributed to factors external to the course itself. Consequently, no significant adverse effects on the participants were observed or reported, satisfying the progression criterion regarding limited efficacy.

### Selv-perceived course effects

The post-course feasibility questionnaire (Q12) included questions regarding self-perceived course effects, as presented in Table 10. When asked whether the course had impacted their daily lives, 14 of the 18 who responded (78%) confirmed that it had. Additionally, 11 participants (61%) perceived that the course had affected how they were feeling. The participants described changes such as eating healthier and increased knowledge, skills, and awareness of what they ate. The social effects were highlighted by the fact that many students enjoyed attending the lessons, which allowed them to cook and eat with others.

**Table 10:** Self-perceived course effects (*n* = 18)

Question	*n* (%^a^)	Examples of changes described
Has the course affected your daily life? - Yes, a lot - Yes, a little - I don’t know - No	5 (28%) 9 (50%) 4 (22%) 0 (0%)	- Try to include more greens when cooking - Easier to get started with cooking - Increased awareness of what I eat - More creative when cooking - Increased confidence when cooking to myself and others - Increased knowledge and skills - Try to follow the plate model - Cook more from scratch and better at hygiene - Eat healthier - Drink more milk - Less energy on course days because cooking is tiring
Has the course affected how you are feeling? - Yes, a lot - Yes, a little - I don’t know - No	3 (17%) 8 (44%) 3 (17%) 4 (22%)	- I notice that the food is more filling and that I feel healthier - I loved attending the lessons. I always came in a good mood that day - I was happy to meet new people and to eat something nice with others - Increased motivation when cooking and eating with others - I spend a little less on food and my meals are somewhat more reasonable [healthy/balanced]

^a^Percentages may not add up to 100% due to rounding. Ten of the 28 participants did not respond. Percentages are calculated based on the number of responses for each question

## Discussion

The aim of this study was to evaluate the feasibility of the *Skills for Life* course. This is one of the few studies that examines the feasibility of a university course aimed at enhancing cooking skills and healthy diets among university students. As the results indicate, all progression criteria were met, allowing the course to be recommended for efficacy testing. It should be noted that the progression criteria were set in conjunction with the reporting of data, and not a priori as recommended [45, 50].

### Acceptability

In our study, acceptability was measured as students’ satisfaction with and perceived suitability of the course. The participants were satisfied with the course and perceived it as suitable, with median acceptability scores ranging from 4.5 to 5 out of 5 (Tables 6 and 7). This might be explained by the extensive user involvement when designing the course, as the course was co-created with students from the target group. A similar course, which focused on a planetary health diet, was recently piloted in Germany [51] with similar acceptability results as those of *Skills for Life*. Although Rosenau and colleagues did not specify co-creation as a way of designing the course, the participants were directly involved in the course by presenting key topics in the seminars and choosing recipes for the cooking sessions [51].

Several *Skills for Life* course participants reported that they enjoyed cooking and eating with others. The interactive nature of the course may have contributed to the high acceptability score, as there was time to discuss and reflect upon relevant topics regarding food and health, prepare food in groups, and enjoy a tasty and nutritious meal together. This may indicate that the participants’ need for relatedness was met, promoted by a sense of belonging within the group and support from both peers and facilitators [37]. This support was sustained regardless of success or failure, such as in the outcomes of their cooked dishes, aligning with one of the previously suggested relatedness-support techniques [46]. Furthermore, by being voluntary, employing informational, non-judgemental and non-controlling language, and providing a meaningful rationale for healthy eating, the course promoted students’ need for autonomy. This aligns with the SDT perspective [37] and previously suggested autonomy-support techniques [46].

### Demand

Demand for the *Skills for Life* course was measured by documenting the number of enrolments, participants, and use of selected intervention activities. Among the 69 students who signed up for the intervention, 28 (41%) attended at least one lesson. Both numbers were well above the progression criteria of at least 30 enrolments and 20 participants. Although an invitation to participate in the course was sent to 1000 first-year undergraduate students, only five participants reported that they learned about the course through an invitation e-mail. The low response rate (0.5%) may be partly explained by the fact that many students do not check their institutional e-mail regularly, and that university students have other priorities.

The focus group discussions with students about the proposed dietary life skills course, conducted prior to the intervention, revealed significant interest among students in attending such an initiative [18]. These undergraduate students represented all the different academic disciplines at UiA but had already been primed by discussing food and health-related topics before discussing the course. The enrolment of 69 students suggests some demand for the course; however, first-year students might not constitute the most appropriate target group. The setting of entering university can be a challenging transition [52], and many new students may not feel that they have the capacity to perform extra tasks, such as attending a cooking course, in addition to their regular studies, work, and leisure activities. Rosenau and colleagues recruited all kinds of students from the entire university, and more than 40% were master’s students [51], illustrating a demand for food or cooking courses among senior students in Germany. As we tailored the course and recruitment to first-year students, other students who saw the course advertisements may have thought that the course was not relevant to them.

The overall attendance rate of 49% among the 28 that attended *Skills for Life* is somewhat lower than we expected. Only seven students (25%) met the 80% attendance requirement necessary to obtain a course certificate. Attendance decreased dramatically after the autumn break, possibly due to increased workload throughout the semester, which made the participants prioritise their regular studies. Mahmoud and colleagues also reported a high drop-out rate from a university cooking course in Canada [53]. In the aforementioned German cooking course, however, 27 out of 35 students (77%) met the attendance requirement of 80% [51]. Notably, there is an important difference between *Skills for Life* and the German course as the latter conferred credits. In line with our previous research, one might assume that courses that confer credits will be of higher priority to students [18].

### Usage of online resources

With respect to the use of online resources, most of the participants reported using the website and watching at least some videos. However, it seems that they prioritised attending the cooking lessons rather than engaging with the website. According to previous research on the flipped classroom teaching model, pre-class videos were well received by students, but they underlined that the videos should be short and engaging [54]. In that study, instructor-made videos received the highest rating compared with videos made by a guest speaker or alternative sources [54]. The *Skills for Life* videos were planned and presented by the instructor, while the IT and Communication departments at UiA assisted by incorporating illustrations, animations, and film clips, and by highlighting text to substantiate and clarify the message and engage the audience. The videos were up to 12 min long, considerably shorter than the maximum length of 20 min previously suggested by students [54]. However, this length might still be too long to engage students in today’s highly digital and dynamic world, especially in a voluntary course without tests, exams, and credits. Despite this, the voluntary nature of the course was an important attribute to avoid adding stress to the participants and to support their need for autonomy, which is consistent with SDT [37]. Allowing participants to make their own choices and set their priorities is a prerequisite for the internalisation of behaviours and promotion of autonomous motivation [37].

The students’ motivation for attending the course might have varied. Some might have been motivated by having free dinners or making new friends, others by becoming better cooks or learning about nutrition. The post-course feasibility survey (Q12) contained a question on why the participants signed up for the course, and the most predominant motivators seemed to be related to the need for competence described in SDT [36, 37]: learning about cooking and a healthy diet and increasing their cooking skills (data not shown). Some also mentioned social aspects, and that the course was free. The social aspects can be connected to the SDT need for relatedness [37]. The reasons for not attending were most often reported to be participation in their regular studies or illness (13 participants answered this question, data not shown).

The fact that none of the participants had listened to the podcast was surprising, indicating that it could have been better advertised or that they were not interested in such a way of acquiring knowledge. Data from the pre-intervention focus group discussions revealed that the course should provide in-depth knowledge and that the students wanted to learn something new that was valuable to their own life and health [18]. This would support the internalisation of motivation and the participants’ need for competence, thereby facilitating behaviour change in accordance with SDT [37]. However, since the *Skills for Life* course was undertaken alongside regular studies, it ought not to take up too much of the participants’ leisure time. One of the barriers to attending *Skills for Life* that emerged from the aforementioned focus group discussions was the perception that the course was too demanding or time-consuming [18].

### Implementation and practicality

The intervention was implemented as planned and proposed. Ten lessons were delivered within normal working hours, in accordance with the progression criteria. Flyers, roll-up, and e-mail invitations were reportedly the most effective recruitment strategies.

In our study, practicality was evaluated by estimating the time and money spent on conducting the course. The resources needed varied from session to session and depended on factors such as how experienced and effective the facilitator was. Overall, 1 to 1.5 h of practical preparation per session would be adequate, in addition to some practical work after each session according to the routines at the teaching kitchen. Some of the lessons lasted slightly longer than the estimated duration of 2 to 2.5 h, especially when few participants attended. As many students did not send a notice when they were prohibited from attending, it was challenging to plan what to cook and how much food to buy. The students who attended were, however, happy to eat their fill and to take leftovers home. In this way, food waste was avoided. In particular, Group 2 experienced low attendance, often resulting in lessons not concluding on time because the workload was distributed among a limited number of participants. The mean per-person cost of NOK 71 could have been reduced if the attendance rate had been higher because much of the food was left after the meals. The kitchen facilities were available for free, and other expenses such as electricity, printed handouts, instructor pay, etc., were covered by the university.

Two undergraduate students in nutrition helped to prepare and conduct several of the cooking lessons. Other studies have also utilised students as course deliverers [53] or facilitators in cooking sessions [55]. This is a way of reducing staff workload and salary expenses, in addition to enhancing learning outcomes and providing valuable work experience for student educators or facilitators. One of the participants in the study by Mahmoud et al. reported that she enjoyed talking to peer educators because they were the same age as herself, which made it easier to ask them questions [53]. The use of two undergraduate students as facilitators in *Skills for Life*, in addition to an early career course leader, may have contributed to the high level of course satisfaction.

### Limited efficacy

In accordance with the progression criteria, no significant adverse effects on participants were reported or detected. An interesting finding was the increased post-course legume score, possibly caused by the consumption of legumes during the course, combined with the focus on (canned) legumes as a cheap, quick, sustainable, and healthy alternative to meat. However, owing to the small sample size and lack of statistical power, the implied improvements in diet quality and food habits cannot be confirmed statistically. Furthermore, there was no control group. This means that possible dietary improvements might have been caused by other factors such as seasonal variations or the establishment of autonomous eating habits during the first months as university students. The baseline questionnaire was completed in late August, reflecting food intake during the summer break and semester start. This period may include the transition from living with parents to moving away to study, a phase previously associated with deteriorated eating habits [56]. The post-course questionnaire was completed in late November, when the exam period and Christmas were approaching. This may negatively affect diet because of increased intake of Christmas cakes and sweets and because of exam stress. Pope and colleagues used a randomised controlled design to evaluate a cooking course aiming to improve university students’ ability to plan, procure, and prepare food (food agency) [57]. This course included six weekly cooking classes that induced significant improvements in food agency skills. No improvement in diet quality was observed; rather, the opposite was reported. The authors postulated that this might be, similar to the *Skills for Life* setting, due to the flow of the semester and stressing exam periods where healthy eating may have a low priority [57]. In contrast, fourteen *Skills for Life* participants reported self-perceived course effects, such as healthier eating habits and increased knowledge, skills, and awareness of their diet. The course period of 12 weeks might not be enough to induce measurable changes in the diet, or the dietary assessment methods used might not have been detailed enough to detect changes. However, increased knowledge, skills, and awareness might improve diet in the long run, in line with a life course approach [9, 10].

The diet quality score in our sample was moderate (mean value 47.2 before the course) and substantially lower than the score reported in another sample of preconception young adults in Norway, where the mean value was 60 [29]. This implies room for improvement in this group, indicating a need to further investigate whether the course can induce long-term effects on diet quality in a larger sample, preferably including a control group to be able to evaluate a causal relationship.

### Reflections on how the intervention could be improved

Regarding the scope of the course, 10 sessions could be too many to include in a busy student schedule. A possible alternative may be to reduce the number of sessions to five or six and prioritise the most important content. This aligns more closely with previous cooking courses in higher education that have demonstrated positive effects [51, 53, 55, 57, 58]. Another option might be to deliver five sessions during the autumn semester and five during the following spring semester. The course was delivered early afternoon, but since many students may attend lectures, group work, or student placement at those time points, offering the course later in the afternoon or early evening might be more appropriate for some students. Investigating whether the course could award credits would also be of interest, as this seemed to be a motivator in our previous research (18). Notably, the cooking course delivered by Rosenau and colleagues [51] awarded credits and had high retention.

Given the low attendance rate, it is likely that regular studies or other activities fulfilled many participants’ needs for autonomy, competence, and relatedness to a greater extent than *Skills for Life*. Qualitative interviews with 10 course participants were conducted post-course, and insights from these interviews will provide further advice on how the intervention can be improved. The interview data will be used for a future publication. Although the acceptability ratings were high across all the lessons, the course could have been further tailored to better meet the students’ needs. This could have included an increased focus on cooking techniques and theoretical inputs during classes and better aligning the course level with participants’ individual cooking skills. Offering a range of recipes from very simple to more advanced in each lesson could have enhanced the promotion of the need for competence. More of the competence-support techniques suggested by Teixeira et al. could have been utilised, such as assisting in setting optimal challenges, clarifying expectations, and exploring ways of dealing with pressure [46]. Considering that cost and time are significant priorities for university students when cooking [19], even simpler, quicker, and cheaper recipes could have been used and distributed. A prior cooking course in higher education supplied recipes with nutritional information for participants to take home [58]. Such a practice could also have been implemented in *Skills for Life*, along with the inclusion of estimated costs and preparation time for each recipe.

#### Strengths and limitations

Bearing in mind that this was a feasibility study, a randomised controlled study should be performed to evaluate course effects before the course can be implemented in other settings. The course was co-created with the target group, strengthening its relevance and potential to be effective [34]. Setting the progression criteria in conjunction with the reporting of data instead of prior to data collection was a limitation, increasing the probability of bias [45, 50]. Additionally, the progression criteria were set without involving the target group, and from a co-creation perspective, this was a limitation [50, 59]. However, following the framework of feasibility studies by Bowen and colleagues [31] was a strength.

Using a validated dietary screener was a strength that enabled us to compare our findings with those from other studies that used the same instrument. Additionally, the screener is quick and easy to complete compared with more comprehensive tools [40], facilitating focused and thorough participant responses. Mistakenly omitting two of the response categories when transmitting the screener to SurveyXact was a limitation, diminishing the reproducibility and reliability of our data on the diet quality score. To prevent this issue in the future, the questionnaire should be thoroughly compared with the original screener by more than one researcher before distribution. Compared with objective measures, self-reported data are susceptible to a greater likelihood of measurement errors [60]. The questions regarding self-perceived course effects could have been formulated in a more neutral way and included a balance of positive, negative, and neutral response options to avoid bias towards positive responses. To mitigate the influence of social desirability bias in the evaluation of both the lessons and the overall course, we explicitly asked the participants to provide their honest opinions.

Multiple measurements for acceptability with a high response rate among those attending the course increased the representativity and validity of the findings. With respect to generalisability, the gender balance (64% women and 36% men) corresponded fairly well to that of UiA in general (59% women and 41% men in 2023). The fact that students from all seven UiA academic disciplines were represented among participants further increased the representativity of our findings.

## Conclusions

*Skills for Life* has proven feasible in a university setting. The participants reported self-perceived course effects, including a healthier diet, increased knowledge of healthy eating, improved cooking skills, and greater awareness of their dietary habits. Additionally, the students noted social benefits and overall satisfaction with the course. However, the challenge of prioritising the course alongside regular studies and work commitment was evident, as reflected by an overall attendance rate of only 49%. Studies with larger sample sizes and control groups are warranted to investigate the effects of such a course. The implementation of a revised *Skills for Life* course in similar settings may improve dietary knowledge, cooking skills, and possibly diet in students to support future health for themselves and the next generation.

## Supplementary Information

Additional file 1. Screenshots from the Skills for Life website.

Additional file 2. Questionnaires.

Additional file 3. Overview of learning objectives and the 10 Skills for Life cooking lessons.

Additional file 4. ’MyFoodMonth 1.1’ Diet Quality Score scorings.

## Data Availability

The datasets generated and analysed during the current study will be available in the DATAVERSE repository upon acceptance.

## Abbreviations

DOHaD: Developmental origins of health and disease
IQR: Interquartile range
Q1 − Q13: Questionnaire numbers 1 − 13
SD: Standard deviation
SDT: Self-determination theory
UiA: University of Agder
WHO: World Health Organization
