# Supplementary material for: Feasibility of a dietary life skills course aimed at fostering cooking skills and a healthy diet among university students
Source: Pilot Feasibility Stud. 2025 Jul 17;11:100. doi: 10.1186/s40814-025-01680-y (PMC12273360; doi:10.1186/s40814-025-01680-y)
Supplement: Supplementary file 1 — Additional file 1. Screenshots from the Skills for Life website. [file 40814_2025_1680_MOESM1_ESM.pdf]

Additional file 1: Screenshots from the Skills for Life website

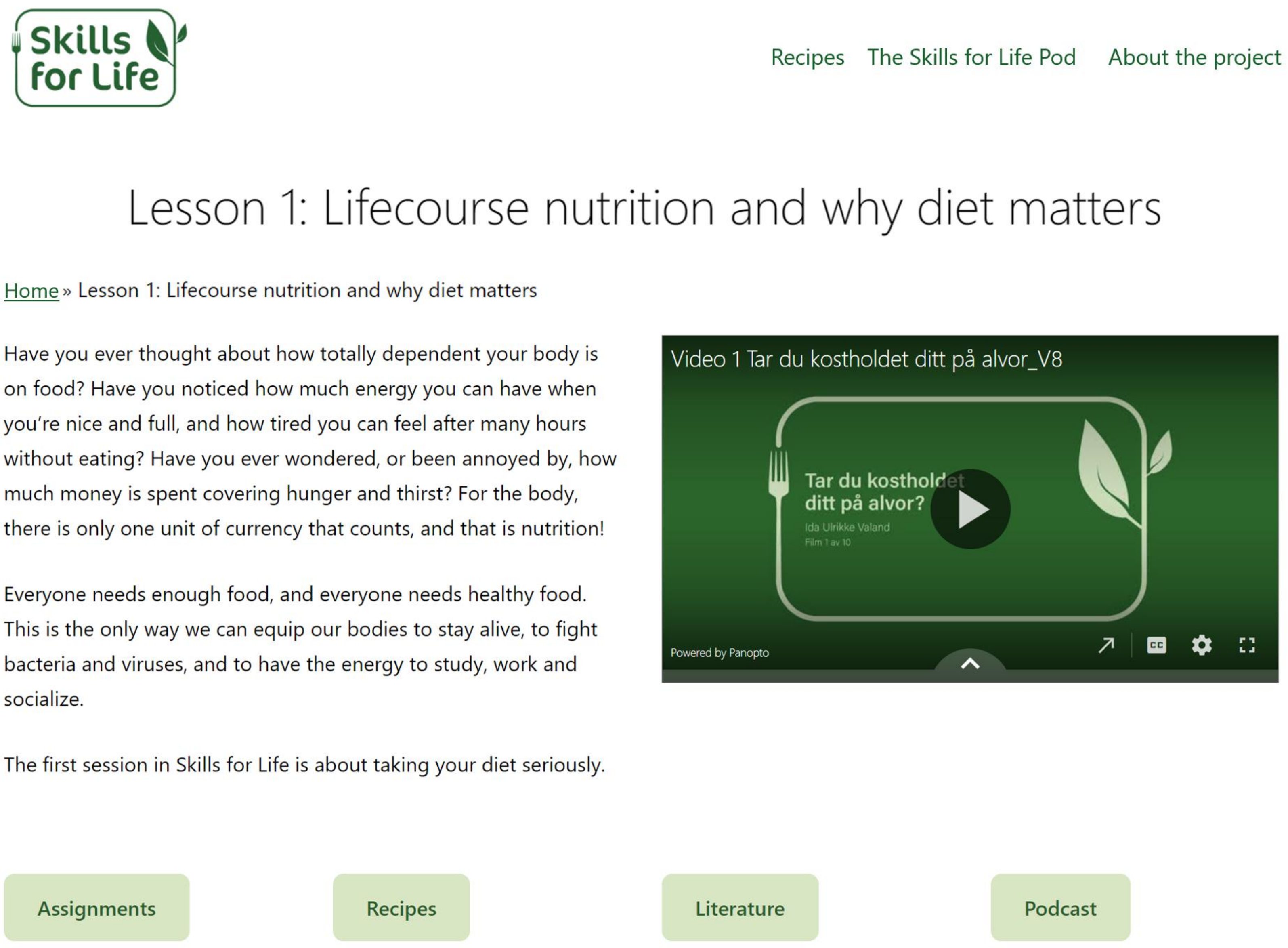

Figure 1. Screenshot from the website (skillsforlife.uia.no, lesson 1, translated to English)

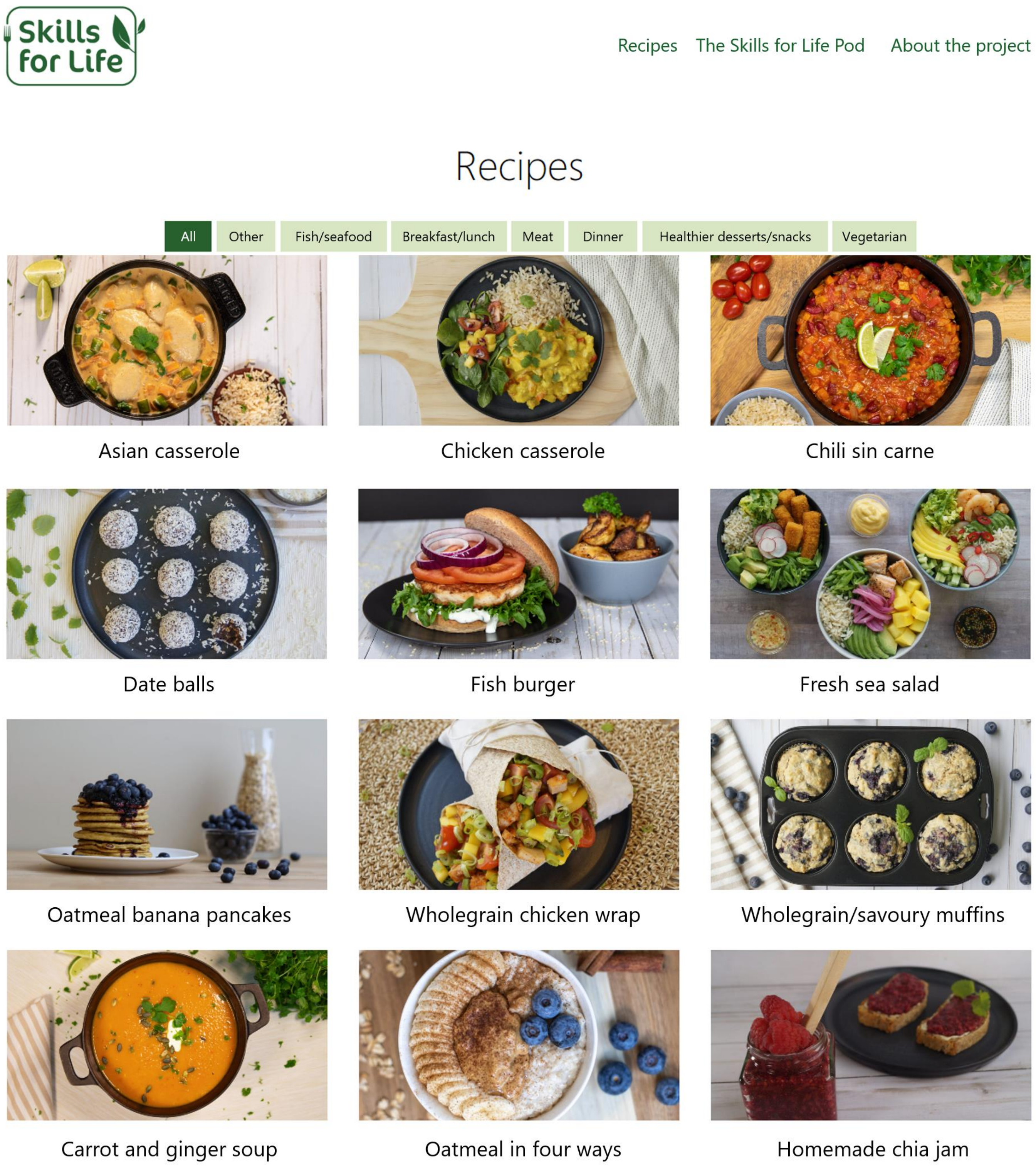

Figure 2. Screenshot from the website (skillsforlife.uia.no, some of the recipes, translated to English)
