# Supplementary material for: Feasibility of a dietary life skills course aimed at fostering cooking skills and a healthy diet among university students
Source: Pilot Feasibility Stud. 2025 Jul 17;11:100. doi: 10.1186/s40814-025-01680-y (PMC12273360; doi:10.1186/s40814-025-01680-y)
Supplement: Supplementary file 2 — Additional file 2. Questionnaires. [file 40814_2025_1680_MOESM2_ESM.pdf]

Additional file 2: Questionnaires

Questionnaire 1 – screenshots from the original version in Norwegian

Dette spørreskjemaet tar omkring 20-30 minutter å fylle ut.

Tusen takk for at du bidrar til viktig forskning!

Hva er din alder (fyll inn antall år)?

____

Hva er ditt kjønn?

- ☐ Mann
- ☐ Kvinne
- ☐ Annet/vil ikke oppgi

Hvilket studieprogram går du på?

____

Hvilket studieår er du i nå?

____

Har du studert før? I så fall, hva?

- ☐ Ja (fyll inn hva du har studert før)__________
- ☐ Nei

Bor du sammen med andre? I så fall, hvem?

- ☐ Ja, foreldre
- ☐ Ja, samboer/ektefelle
- ☐ Ja, i kollektiv
- ☐ Ja, annet (beskriv)__________
- ☐ Nei

Har du hovedansvar for matlagingen hjemme?

- ☐ Ja
- ☐ Nei
- ☐ Ansvaret er delt

Har du ansvar for barn hjemme (egne barn/bonusbarn)?

- ☐ Ja
- ☐ Nei

I hvilken grad opplever du at ditt kjøkken/tilgang på kjøkken er begrensende for kosthold/matlaging?

- ☐ I stor grad
- ☐ I liten grad
- ☐ Ikke i det hele tatt

Hvor ofte?

	Aldri	Mindre enn 1 gang per uke	1 g/uke	2 g/uke	3 g/uke	4 g/uke	5 g/uke	6 g/uke	Hver dag
...Kutter du opp grønnsaker?	☐	☐	☐	☐	☐	☐	☐	☐	☐
...Kutter du opp frukt?	☐	☐	☐	☐	☐	☐	☐	☐	☐
...Lager du middag fra bunnen?	☐	☐	☐	☐	☐	☐	☐	☐	☐

Har du inntekt utover studielån/stipend?

- ☐ Ja
- ☐ Nei

Hvor enig er du i følgende utsagn? "Jeg har nok penger til å kjøpe sunn mat"

- ☐ Svært uenig
- ☐ Uenig
- ☐ Nøytral
- ☐ Enig
- ☐ Svært enig

Kort spørreskjema om kostholdet ditt

Spørsmålene gjelder utvalgte mat- og drikkevarer den siste måneden, det vil si de siste 30 dagene.

Angi hvor ofte du har spist og drukket de nevnte mat- og drikkevarene til måltider, mellommåltider eller som snacks når du har vært hjemme, på farten, på kafé, eller hvor som helst.

Hvor ofte spiste/drakk du følgende siste måned?

	6+ ganger per dag	4-5 ganger per dag	2-3 ganger per dag	1 gang per dag	5-6 ganger per uke	2-4 ganger per uke	1 gang per uke	Sjelden/aldri
Søte og halvsøte frokostblandinger (f.eks. Special K, Honnikorn)	☐	☐	☐	☐	☐	☐	☐	☐
Usøtede frokostblandinger (f.eks. 4-Korn, havregryn/havregrøt, Go'dag og Weetabix)	☐	☐	☐	☐	☐	☐	☐	☐
Grovt brød/knekkebrød/rundstykke (>50 % grovt)	☐	☐	☐	☐	☐	☐	☐	☐
Fiskepålegg (f.eks. makrell i tomat)	☐	☐	☐	☐	☐	☐	☐	☐
Hvitost (alle typer)	☐	☐	☐	☐	☐	☐	☐	☐
Brunost/prim	☐	☐	☐	☐	☐	☐	☐	☐
Yoghurt, skyr o.l. (alle typer)	☐	☐	☐	☐	☐	☐	☐	☐
Kumelk (alle typer)	☐	☐	☐	☐	☐	☐	☐	☐
Plantebasert melk (alle typer)	☐	☐	☐	☐	☐	☐	☐	☐
Juice/smoothie (ikke nektar)	☐	☐	☐	☐	☐	☐	☐	☐
Frukt og bær, inkl. ferske, frosne og hermetiske (ikke juice eller smoothie)	☐	☐	☐	☐	☐	☐	☐	☐
Usaltede nøtter og frø	☐	☐	☐	☐	☐	☐	☐	☐

Hvor ofte spiste/drakk du følgende siste måned?

	6+ ganger per dag	4-5 ganger per dag	2-3 ganger per dag	1 gang per dag	5-6 ganger per uke	2-4 ganger per uke	1 gang per uke	Sjelden/aldı
Grønnsaker, inkl. salat, kål, gulrot, grønne bønner osv. (ikke potet og søtpotet)	☐	☐	☐	☐	☐	☐	☐	☐
Bønner, linser, kikerter, erter (ikke grønne bønner)	☐	☐	☐	☐	☐	☐	☐	☐
Friterte poteter/søtpoteter (f.eks. pommes frites, røstipoteter)	☐	☐	☐	☐	☐	☐	☐	☐
Andre poteter/søtpoteter (feks. bakt, kokt, most)	☐	☐	☐	☐	☐	☐	☐	☐
Fullkorn-middagsprodukter (f.eks. byggris, fullkonspasta, couscous)	☐	☐	☐	☐	☐	☐	☐	☐
Pizza (alle typer)	☐	☐	☐	☐	☐	☐	☐	☐
Tomatsaus, inkl. saus/salsa til taco, ketchup, til pasta o.l. (ikke pizza)	☐	☐	☐	☐	☐	☐	☐	☐
Plantebaserte ferdigprodukter (alle typer kjøtterstatning)	☐	☐	☐	☐	☐	☐	☐	☐
Rødt kjøtt, oppmalt eller stykker (storfe, lam/sau, svin, kje/geit)	☐	☐	☐	☐	☐	☐	☐	☐
Bearbeidet kjøtt (f.eks. bacon, pålegg, pølser)	☐	☐	☐	☐	☐	☐	☐	☐
Fet fisk og fiskeprodukter (f.eks. laks, makrell)	☐	☐	☐	☐	☐	☐	☐	☐
Mager fisk og fiskeprodukter (f.eks. torsk, sei)	☐	☐	☐	☐	☐	☐	☐	☐

Hvor ofte spiste/drakk du følgende siste måned?

	6+ ganger per dag	4-5 ganger per dag	2-3 ganger per dag	1 gang per dag	5-6 ganger per uke	2-4 ganger per uke	1 gang per uke	Sjelden/aldı
Salt snacks (f.eks. popcorn, chips, salte nøtter)	☐	☐	☐	☐	☐	☐	☐	☐
Godteri, inkl. sjokolade	☐	☐	☐	☐	☐	☐	☐	☐
Vafler, boller, kake, kjeks o.l.	☐	☐	☐	☐	☐	☐	☐	☐
Iskrem, panna cotta, pudding, mousse o.l.	☐	☐	☐	☐	☐	☐	☐	☐
Brus, saft og nektar med sukker	☐	☐	☐	☐	☐	☐	☐	☐
Energidrikker med sukker (f.eks. Gatorade, Red Bull)	☐	☐	☐	☐	☐	☐	☐	☐
Kaffe/te/iskaffe/iste med sukker/sirup/honning	☐	☐	☐	☐	☐	☐	☐	☐
Alkoholholdig drikke	☐	☐	☐	☐	☐	☐	☐	☐
Vann	☐	☐	☐	☐	☐	☐	☐	☐

Har du tatt kosttilskudd som vitaminer, proteintilskudd o.l.?

- ☐ Nei
- ☐ Ja

Hvis ja på forrige spørsmål; hva og hvor ofte?

Hvor ofte pleier du å spise følgende måltider i løpet av en uke?

	7 ganger i uken	6 ganger i uken	5 ganger i uken	4 ganger i uken	3 ganger i uken	2 ganger i uken	1 gang i uken	Sjelden/aldri
Frokost	☐	☐	☐	☐	☐	☐	☐	☐
Formiddagsmat/lunsj	☐	☐	☐	☐	☐	☐	☐	☐
Middag	☐	☐	☐	☐	☐	☐	☐	☐
Kveldsmat	☐	☐	☐	☐	☐	☐	☐	☐

Hvor ofte pleier du å spise mellommåltider/snacks?

- ☐ 6+ ganger per dag
- ☐ 4-5 ganger per dag
- ☐ 2-3 ganger per dag
- ☐ 1 gang per dag
- ☐ 5-6 ganger per uke
- ☐ 2-4 ganger per uke
- ☐ 1 gang per uke
- ☐ Sjelden/aldri

Jeg unngår enkelte mat- og drikkevaner på grunn av...:

	Nei, aldri	Av og til	Ja, alltid
...allergi(er) eller intoleranse(r)	☐	☐	☐
...helsen min	☐	☐	☐
...religionen min	☐	☐	☐
...vekten min	☐	☐	☐
...klimahensyn	☐	☐	☐
...dyrevelferd	☐	☐	☐
...veganisme	☐	☐	☐
...andre grunner enn de som er nevnt (beskriv under)	☐	☐	☐

Beskriv mat- og drikkevarene du unngår (hvis aktuelt):

Andre grunner til at du unngår enkelte mat- og drikkevaner (hvis aktuelt):

Kommentarer relatert til mitt kosthold (hvis aktuelt):

Spørreskjema om matkompetanse

Her kommer en rekke utsagn eller påstander relatert til mat og kosthold. Kryss av for svaralternativet som passer best.

	Svært uenig	Uenig	Nøytral	Enig	Svært enig
Sammenlignet med andre daglige gjøremål, opptar matinnkjøp for mye av tiden min	☐	☐	☐	☐	☐
Sammenlignet med andre daglige gjøremål, opptar matlaging eller tilberedning av mat for mye av tiden min	☐	☐	☐	☐	☐
Jeg får ofte for lite tid til å kjøpe eller lage maten jeg ville foretrukket	☐	☐	☐	☐	☐
Sammenlignet med andre gjøremål, opptar spising for mye av tiden min	☐	☐	☐	☐	☐
Sammenlignet med andre daglige utgifter, opptar mat for mye av budsjettet mitt	☐	☐	☐	☐	☐
Jeg prøver alltid å sette av nok penger til mat for meg selv og/eller de jeg har ansvar for	☐	☐	☐	☐	☐
Når jeg har lite penger, prioriterer jeg mat basert på helse	☐	☐	☐	☐	☐
Når jeg har lite penger, prioriterer jeg mat basert på smak	☐	☐	☐	☐	☐
Når jeg har lite penger, prioriterer jeg mat basert på pris	☐	☐	☐	☐	☐

	Aldri	Sjelden	Noen ganger	Ofte	Alltid
Jeg planlegger fremover (for eksempel for dagen eller uka) når jeg tilbereder min egen mat	☐	☐	☐	☐	☐
Jeg planlegger hva jeg skal kjøpe før jeg går for å handle mat	☐	☐	☐	☐	☐

	Svært uenig	Uenig	Nøytral	Enig	Svært enig
Jeg er i stand til å tilpasse planene mine for hva jeg skal spise, selv om omstendighetene mine endrer seg	☐	☐	☐	☐	☐
Jeg er i stand til å tilpasse planene mine for hva jeg skal spise, selv om maten jeg planlegger å spise ikke er tilgjengelig	☐	☐	☐	☐	☐

	Svært uenig	Uenig	Nøytral	Enig	Svært enig
Når jeg kjøper mat fra en restaurant, kafe, eller takeaway, vet jeg hvor mye penger jeg bruker i en gjennomsnittlig uke	☐	☐	☐	☐	☐
Når jeg kjøper mat fra en restaurant, kafe, eller takeaway, sammenligner jeg priser før jeg kjøper mat	☐	☐	☐	☐	☐
Når jeg kjøper mat fra en restaurant, kafe, eller takeaway, prøver jeg å få den beste maten for den beste prisen	☐	☐	☐	☐	☐
Når jeg kjøper mat fra en restaurant, kafe, eller takeaway, sammenligner jeg priser mellom liknende produkter for å få best mulig verdi for pengene	☐	☐	☐	☐	☐
Når jeg kjøper mat fra en restaurant, kafe, eller takeaway, planlegger jeg å benytte meg av kampanjer	☐	☐	☐	☐	☐

	Svært uenig	Uenig	Nøytral	Enig	Svært enig
Når jeg handler mat, sammenligner jeg priser før jeg kjøper mat	❑	❑	❑	❑	❑
Når jeg handler mat, prøver jeg å få den beste maten for den beste prisen	❑	❑	❑	❑	❑
Når jeg handler mat, sammenligner jeg priser mellom liknende produkter for å få best mulig verdi for pengene	❑	❑	❑	❑	❑
Når jeg handler mat, planlegger jeg å benytte meg av kampanjer	❑	❑	❑	❑	❑
Når jeg handler mat, vet jeg hvor mye jeg bruker i en gjennomsnittlig uke	❑	❑	❑	❑	❑

	Svært uenig	Uenig	Nøytral	Enig	Svært enig
Selv om jeg ikke har min vanlige mengde tid tilgjengelig, kan jeg fortsatt spise den maten jeg foretrekker	❑	❑	❑	❑	❑
Selv om jeg ikke har det vanlige kjøkkenutstyret mitt, kan jeg fortsatt spise den maten jeg foretrekker	❑	❑	❑	❑	❑
Selv om jeg er sulten, lager jeg maten jeg hadde planlagt	❑	❑	❑	❑	❑
Jeg balanserer næringsbehovet mitt med tiden, pengene, ferdighetene og kjøkkenutstyret jeg har	❑	❑	❑	❑	❑

	Aldri	Sjelden	Noen ganger	Ofte	Alltid
Når jeg spiser ute, er det viktig for meg at jeg kan finne maten jeg foretrekker	❑	❑	❑	❑	❑
Når jeg handler mat på et kjent sted, finner jeg maten jeg foretrekker å spise	❑	❑	❑	❑	❑
Når jeg handler mat på et nytt sted, finner jeg maten jeg foretrekker å spise	❑	❑	❑	❑	❑
Når jeg kjøper mat fra en kjent restaurant, kafé eller takeaway, finner jeg maten jeg foretrekker å spise	❑	❑	❑	❑	❑
Når jeg kjøper mat fra en ny restaurant, kafé eller takeaway, finner jeg maten jeg foretrekker å spise	❑	❑	❑	❑	❑
Jeg finner maten jeg har råd til	❑	❑	❑	❑	❑

Ingrediensene i ferdigpakkede/emballerte matvarer er viktige for meg når jeg skal bestemme meg for hvilken mat jeg skal kjøpe

❑ Ikke viktig i det hele tatt

❑ Litt viktig

❑ Nøytral

❑ Veldig viktig

❑ Ekstremt viktig

	Svært uenig	Uenig	Nøytral	Enig	Svært enig
Jeg vet hvordan jeg finner informasjon om hvordan fersk mat dyrkes og produseres					
Jeg sammenligner innhold av enten kalorier, fett, sukker eller salt på matvarer for å bestemme meg for hva jeg skal kjøpe					
Jeg vet hvor jeg kan finne informasjon om den miljømessige og etiske påvirkningen av ulike matvarer					
Når jeg handler mat, vet jeg hvordan maten min oppbevares før jeg kjøper den					
Jeg synes det er lett å vite hvilket land ulike matvarer kommer fra					
Jeg prøver å kjøpe fersk mat som for tiden er i sesong i mitt land					
Når jeg spiser ute, kan jeg gjøre en vurdering av ingrediensene i maten jeg har valgt					
Når jeg spiser ute, kan jeg gjøre en vurdering av næringsverdien til maten jeg har valgt					
Jeg vet hvor jeg skal lete etter informasjon om hva som er i ferdigpakket/emballert mat					
Når jeg handler mat, vet jeg hva som er i ferdigpakket/emballert mat jeg kan kjøpe					
Jeg vet hvordan jeg skal oppbevare frukt og grønnsaker for best mulig friskhet og mattrygghet (for at den skal holde seg best mulig)					

	Aldri	Sjelden	Noen ganger	Ofte	Alltid
Jeg er skuffet over utvalget mitt av fersk mat fordi det ikke oppfyller forventningene mine					
Jeg er skuffet over utvalget mitt av bearbeidet/prosessert mat eller ferdigmat fordi det ikke oppfyller forventningene mine					
Jeg er skuffet over utvalget mitt av mat når jeg spiser ute fordi det ikke oppfyller forventningene mine					

	Svært uenig	Uenig	Nøytral	Enig	Svært enig
Jeg er i stand til å tilberede og spise maten jeg foretrekker, selv om noe uventet skjer på kort sikt					
Jeg har ferdighetene som trengs for å tilberede og lage rimelig mat som jeg foretrekker					
Jeg kan tilberede et måltid med ferske eller minimalt bearbeidede ingredienser					
Når jeg tilbereder mat, føler jeg meg trygg på å bytte ut ingredienser med noe annet					
Jeg er i stand til å tilberede maten jeg foretrekker, selv om helsetilstanden min endrer seg					
Jeg vet hvordan jeg finner informasjon om tilberedning av forskjellige matvarer					
Når jeg lager mat, vet jeg hva jeg skal gjøre når noe går galt					

Jeg føler meg sikker på at jeg kan tilberede mat av ingrediensene jeg har for hånden

- ❑ Ikke sikker i det hele tatt
- ❑ Litt sikker
- ❑ Nøytral
- ❑ Moderat sikker
- ❑ Veldig sikker

	Aldri	Sjelden	Noen ganger	Ofte	Alltid
Jeg vasker frukt og grønnsaker før jeg spiser dem	❑	❑	❑	❑	❑
Etter å ha håndtert rått kjøtt, fjærfe (for eksempel kylling) eller fisk, vasker jeg hendene	❑	❑	❑	❑	❑
Etter å ha kuttet rått kjøtt, fjærfe (for eksempel kylling) eller fisk, setter jeg skjærebrettet til side og bruker et annet skjærebrett til annen mat	❑	❑	❑	❑	❑
Før jeg håndterer mat, vasker jeg alltid hendene	❑	❑	❑	❑	❑
Jeg leser informasjonen om oppbevaring og utløpsdato på ferdigpakket/emballert mat	❑	❑	❑	❑	❑
Jeg bruker informasjonen om oppbevaring og utløpsdato på mat når jeg bestemmer meg for om jeg skal spise den	❑	❑	❑	❑	❑

	Ikke sikker i det hele tatt	Litt sikker	Nøytral	Moderat sikker	Veldig sikker
For å forhindre matforgiftning bør fryserens temperatur være -18 grader Celsius eller kaldere	❑	❑	❑	❑	❑
For å forhindre matforgiftning, bør kjøleskapets temperatur være 4 grader Celsius eller kaldere	❑	❑	❑	❑	❑

Mikroorganismer som forårsaker matforgiftning vokser i temperaturer mellom 5 og 60 grader Celsius

- ❑ Svært uenig
- ❑ Uenig
- ❑ Nøytral
- ❑ Enig
- ❑ Svært enig

	Svært uenig	Uenig	Nøytral	Enig	Svært enig
Jeg vet hvilken mat jeg skal spise for å holde meg frisk	☐	☐	☐	☐	☐
Å spise mer frukt og grønnsaker reduserer risikoen for hjertesykdom	☐	☐	☐	☐	☐
Å spise mat med mye mettet fett øker risikoen for hjerte- og karsykdommer	☐	☐	☐	☐	☐
Å spise mat med mye sukker øker risikoen for tannråte	☐	☐	☐	☐	☐
Å spise mat med mye salt øker risikoen for høyt blodtrykk	☐	☐	☐	☐	☐
Å spise mer melk, yoghurt og ost reduserer risikoen for svake bein (skjør beinbygning)	☐	☐	☐	☐	☐
Hva slags mat jeg spiser påvirker helsen min	☐	☐	☐	☐	☐
Hva slags mat jeg spiser påvirker velværet mitt (hvordan jeg har det)	☐	☐	☐	☐	☐

Følelsene mine påvirker matvalgene mine

- ☐ Aldri
- ☐ Sjelden
- ☐ Noen ganger
- ☐ Ofte
- ☐ Alltid

De norske kostrådene anbefaler...

	Ikke sikker i det hele tatt	Litt sikker	Nøytral	Moderat sikker	Veldig sikker
...å spise grønnsaker hver dag	☐	☐	☐	☐	☐
...å spise frukt hver dag	☐	☐	☐	☐	☐
...å begrense inntak av sukkerholdig mat og drikke	☐	☐	☐	☐	☐
...å spise fullkorn hver dag	☐	☐	☐	☐	☐
...å drikke vann hver dag	☐	☐	☐	☐	☐
...å begrense inntak av bearbeidet kjøtt	☐	☐	☐	☐	☐
...å begrense inntak av mat med mettet fett	☐	☐	☐	☐	☐
...å spise magre meieriprodukter hver dag	☐	☐	☐	☐	☐
...å begrense inntak av salt og mat med mye salt	☐	☐	☐	☐	☐
...å spise minst 5 porsjoner frukt og grønnsaker hver dag	☐	☐	☐	☐	☐
En porsjon grønnsaker tilsvarer en gulrot (100 g)	☐	☐	☐	☐	☐
En porsjon frukt er en middels stor frukt (100 g)	☐	☐	☐	☐	☐
Fire ekstra grove brødskiver er nok til å dekke anbefalt inntak av fullkorn	☐	☐	☐	☐	☐
En porsjon magre meieriprodukter er f.eks. et glass lettmelk (2 dL) eller 2 osteskiver	☐	☐	☐	☐	☐

	Svært uenig	Uenig	Nøytral	Enig	Svært enig
Jeg gjør en bevisst innsats for å prøve å spise sunt	☐	☐	☐	☐	☐
Når jeg bestemmer meg for hva jeg skal spise, tenker jeg på sunne valg	☐	☐	☐	☐	☐
Jeg bruker næringsdeklarasjonen på ferdigpakkede/emballerte matvarer for å bestemme meg for hva jeg skal kjøpe	☐	☐	☐	☐	☐

	Svært uenig	Uenig	Nøytral	Enig	Svært enig
Jeg er komfortabel med å spise sammen med andre mennesker	☐	☐	☐	☐	☐
Å spise sammen med andre handler om mer enn bare mat	☐	☐	☐	☐	☐
Å spise bringer mennesker sammen på en hyggelig måte	☐	☐	☐	☐	☐

Når jeg spiser sammen med andre, er det viktig for meg å sette meg ned og spise ved et bord

☐ Ikke viktig i det hele tatt
☐ Litt viktig
☐ Nøytral
☐ Moderat viktig
☐ Ekstremt viktig

Jeg spiser sammen med andre mennesker

☐ Aldri
☐ Sjelden
☐ Noen ganger
☐ Ofte
☐ Alltid

	Svært uenig	Uenig	Nøytral	Enig	Svært enig
Mat er en sentral del av hvordan jeg får venner eller danner relasjoner med andre mennesker	☐	☐	☐	☐	☐
Mat er en sentral del av hvordan jeg feirer anledninger eller kulturelle begivenheter sammen med andre mennesker	☐	☐	☐	☐	☐

Tusen takk!

Questionnaire 1 – English translation

This questionnaire takes about 20-30 minutes to complete.

Thank you for contributing to important research!

What is your age (fill in the number of years)?

____

What is your gender?

- ☐ Male
- ☐ Female
- ☐ Other/prefer not to say

Which study programme are you enrolled in?

____

What study year are you currently in?

____

Have you studied before? If so, what?

- ☐ Yes (fill in what you have studied before) ____________
- ☐ No

Do you live with others? If so, who?

- ☐ Yes, with parents
- ☐ Yes, with partner/spouse
- ☐ Yes, in shared accommodation
- ☐ Yes, other (please describe)
- ☐ No

Do you have primary responsibility for cooking at home?

- ☐ Yes
- ☐ No
- ☐ Shared responsibility

Do you have children or stepchildren you are responsible for at home?

☐ Yes
☐ No

To what extent do you feel that your kitchen/access to a kitchen is limiting for diet/cooking?

☐ To a large extent
☐ To a small extent
☐ Not at all

How often?

	Never	Less than once per week	Once per week	Twice per week	3 times per week	4 t/w	5 t/w	6 t/w	Every day
...do you cut vegetables	☐	☐	☐	☐	☐	☐	☐	☐	☐
...do you cut fruits	☐	☐	☐	☐	☐	☐	☐	☐	☐
...do you cook dinner from scratch	☐	☐	☐	☐	☐	☐	☐	☐	☐

Do you have income in addition to student loans/grants?

☐ Yes
☐ No

How much do you agree with the following statement? 'I have enough money to buy healthy food.'

☐ Strongly disagree
☐ Disagree
☐ Neutral
☐ Agree
☐ Strongly agree

Short questionnaire about your diet[1]

The questions apply to selected food and beverage items consumed during the last month, that is, the last 30 days. Enter how often you ate or drank the foods and beverages listed below either at meals or as a snack (in-between-meal), at home, on the go, in a café, or anywhere.

[1] This questionnaire is retrieved (with appropriate modifications) from Salvesen L, Wills AK, Øverby NC, Engeset D, Medin AC. Relative validity of a non-quantitative 33-item dietary screener with a semi-quantitative food frequency questionnaire among young adults. Journal of Nutritional Science. 2023;12:e72.

1. How often did you eat/drink the following items the last month? (Enter once per row)	Times per day				Times per week			Rarely /never
	6+	4-5	2-3	1	5-6	2-4	1	
**Cereal and porridge**								
Sweetened (e.g., Special K, Corn Flakes with honey)								
Unsweetened (e.g., 4-Korn muesli, oatmeal, Go'dag muesli, and Weetabix)								
**Whole grain bread, crispbread, rolls (>50% whole grain)**								
**Fish spread (e.g., mackerel in tomato sauce)**								
**White cheese (all types)**								
**Whey cheese**								
**Yoghurt, skyr (all types)**								
**Cow's milk (all types)**								
**Plant-based milk (all types)**								
**Juice/smoothie (not nectar)**								
**Fruit and berries, including fresh, frozen, and canned (not juice or smoothie)**								
**Unsalted nuts and seeds**								
**Vegetables, including salad, cabbage, carrot, green beans, etc. (not potatoes or sweet potatoes)**								
**Beans, lentils, chickpeas, peas (not green beans)**								
**Fried potatoes / sweet potatoes (e.g., fries, roast potatoes)**								
**Potatoes / sweet potatoes, other (e.g., baked, boiled, mashed)**								
**Whole grain dinner products (e.g., barley, pasta, couscous)**								
**Pizza (all types)**								
**Tomato sauce, including sauce/salsa for tacos, ketchup, pasta, etc. (not pizza)**								
**Plant-based substitutes (all types of meat substitutes)**								
**Red meat, minced or cuts (beef, lamb/mutton, pork, kid)**								
**Processed meat (e.g., bacon, spread, sausage)**								
**Fatty fish and fish products (e.g., salmon, mackerel)**								
**Lean fish and fish products (e.g., cod, pollock)**								
**Salty snacks (e.g., popcorn, chips, salty nuts)**								
**Candy, including chocolate**								
**Waffles, buns, cake, biscuits etc.**								
**Ice cream, panna cotta, pudding, mousse, etc.**								
**Sugar-sweetened beverages**								
**Sugar-sweetened energy drinks (e.g., Gatorade, Red Bull)**								
**Coffee / tea / iced coffee / iced tea with sugar/syrup/honey**								
**Alcoholic beverages**								
**Water**								

2. Have you taken any supplements such as vitamins, protein supplement etc.?
No ☐<br>Yes ☐
If yes; what and how often?

3. How often do you usually eat the following meals per week? (Enter once per row)								
	Times per week							Rarely/ never
	7	6	5	4	3	2	1	
**Breakfast**								
**Lunch**								
**Dinner**								
**Supper**								
	Times per day				Times per week			Rarely/ never
	6+	4-5	2-3	1	5-6	2-4	1	
**Snack (in-between-meals)**								

4. I avoid consuming certain foods and beverages because of …: (Enter once per row)			
	No, never	Sometimes	Yes, always
… allergies or intolerance(s)			
… my health			
… my religion			
… my weight			
… climate considerations			
… animal welfare			
… veganism			
… other reasons than those above (describe below)			
**Describe the foods and beverages you avoid (if applicable):**			
**Other reasons why you avoid consuming certain foods and beverages (if applicable):**			

Comments related to my diet (if applicable):

Questionnaire on food competence

Here you'll find a series of statements related to food and diet. Tick the box for the answer option that best suits you.

The original version of this questionnaire (in English) can be found in Thompson C, Byrne R, Adams J, Vidgen HA. Development, validation and item reduction of a food literacy questionnaire (IFLQ-19) with Australian adults. International Journal of Behavioral Nutrition and Physical Activity. 2022;19(1):113.

Questionnaire 2 – screenshots from the original version in Norwegian

Dette er en kort spørreundersøkelse hvor jeg ønsker din ærlige tilbakemelding
på første kjøkkenøkt med tema: Tar du kostholdet på alvor?
Undersøkelsen tar ca. 2-5 minutter.

Tusen takk for innspillene!

Tema for denne økta er viktig for meg å lære om

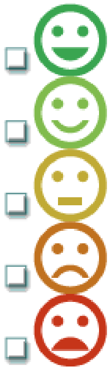

Jeg likte denne økta

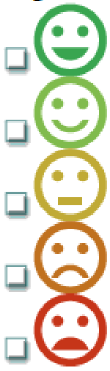

Jeg lærte noe nytt fra denne økta

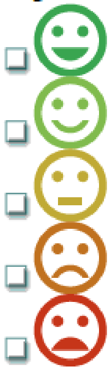

Andre studenter ville ha likt denne økta

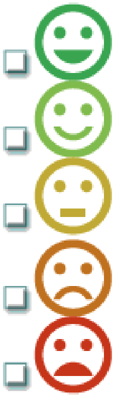

Det er viktig for studenter å delta i denne økta

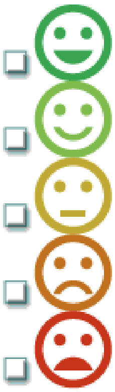

Gruppearbeidet, oppgavene og matlagingen var passende, lærerike og nyttige

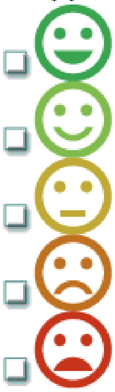

Har du utfyllende kommentarer eller endringsforslag?

Questionnaire 2 – English translation

This is a short survey where I would like your honest feedback on the first kitchen session with the topic: Nutrition from a life course perspective and why diet matters.
The survey takes 2-5 minutes.
Thank you very much for your input!

The topic of this lesson is important for me to learn about

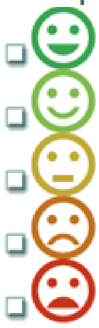

I liked this lesson

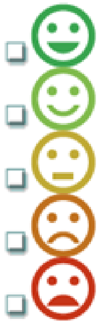

I learned something new from this lesson

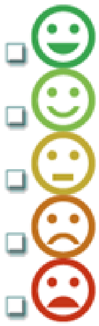

Other students would like this lesson

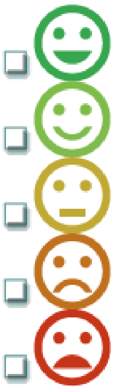

It is important for students to attend this lesson

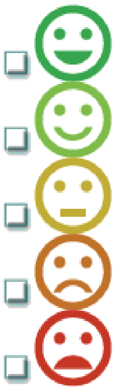

The group work, activities and cooking were appropriate, educational, and useful

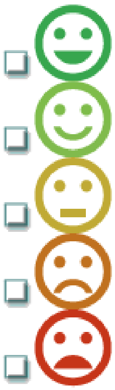

Do you have any additional comments or suggestions for changes?

Questionnaire 12 – screenshots from the original version in Norwegian

Dette er en spørreundersøkelse hvor vi ønsker din ærlige tilbakemelding på **hele Skills for Life-kurset,** altså alle de 10 praktiske øktene på kjøkkenet og tilhørende innhold på Skills for Life-nettsida (inkludert filmer, oppgaver, oppskrifter, litteraturtips og Skills for Life-podden).
Undersøkelsen tar omkring 5-10 minutter å svare på.

Tusen takk for innspillene dine!

Hvordan fikk du vite om kurset?

- ☐ Jeg så en reklame på Facebook
- ☐ Jeg så reklamefilmen på en av skjermene på UiA
- ☐ Jeg så/fikk en flyer
- ☐ Jeg så roll-upen i Vrimlehallen
- ☐ Jeg la merke til standen på Plenen under åpningsseremonien
- ☐ Foreleseren min informerte om kurset i en forelesning
- ☐ Jeg fikk høre om det fra en venn/bekjent
- ☐ Jeg fikk høre om Skills for Life på Bli student!-kurset som ble arrangert av UiA PULS i forbindelse med studiestart
- ☐ Annet, fyll ut__________

Hva fikk deg til å melde deg på Skills for Life?

Hvor mange av de 10 kjøkkenøktene har du deltatt på? Om du ikke husker, angi omtrentlig antall.

- ☐ 0
- ☐ 1
- ☐ 2
- ☐ 3
- ☐ 4
- ☐ 5
- ☐ 6
- ☐ 7
- ☐ 8
- ☐ 9
- ☐ 10

Hva fikk deg til å komme på kursøktene?

Hva gjorde at du ev. ikke kom på kursøktene?

Vi vil gjerne at du vurderer utsagnene nedenfor ved å krysse av på fjeset du synes passer best:

Tema for Skills for Life-kurset er viktig for meg å lære om

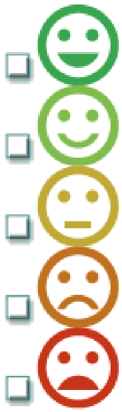

Jeg likte Skills for Life-kurset

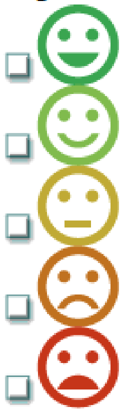

Jeg lærte noe nytt på Skills for Life-kurset

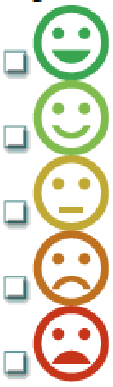

Andre studenter ville ha likt Skills for Life-kurset

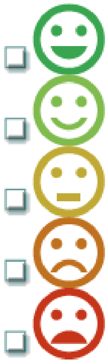

Det er viktig for studenter å delta på Skills for Life-kurset

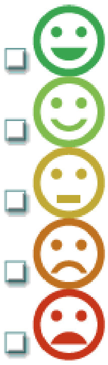

Gruppearbeidet, oppgavene og matlagingen i de praktiske kjøkkenøktene var passende, lærerike og nyttige

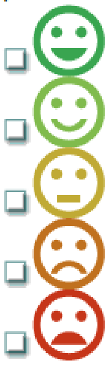

Har du brukt noen av oppskriftene fra Skills for Life hjemme (enten oppskrifter fra de praktiske øktene, eller fra Skills for Life-nettsida)?

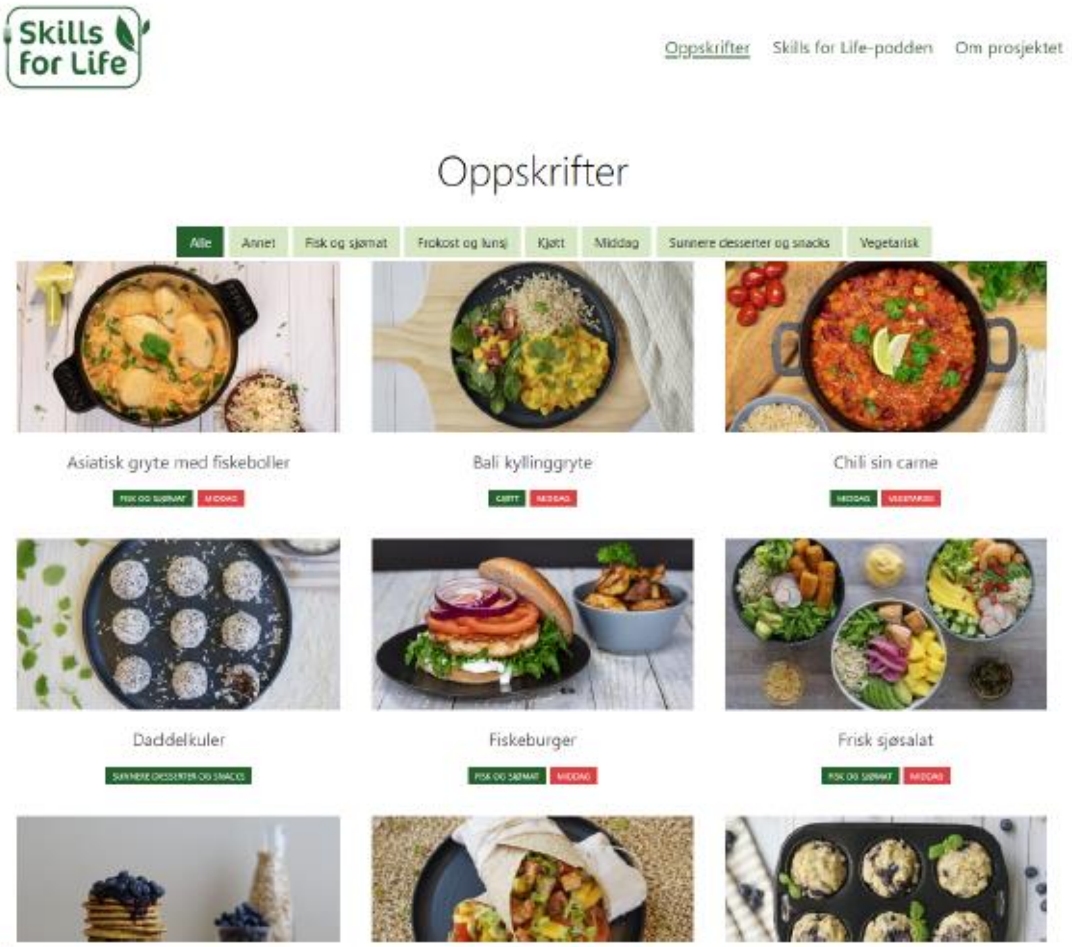

- ☐ Ja, mange
- ☐ Ja, noen
- ☐ Vet ikke
- ☐ Nei

Hvis du har brukt noen av oppskriftene hjemme; hvilke?

Har kurset påvirket hverdagen din?

- ☐ Ja, mye
- ☐ Ja, litt
- ☐ Vet ikke
- ☐ Nei

Hvis kurset har påvirket hverdagen din, hvordan?

Har kurset påvirket hvordan du har det?

- ☐ Ja, mye
- ☐ Ja, litt
- ☐ Vet ikke
- ☐ Nei

Hvis kurset har påvirket hvordan du har det; beskriv endringen:

Har du brukt Skills for Life-nettsida?

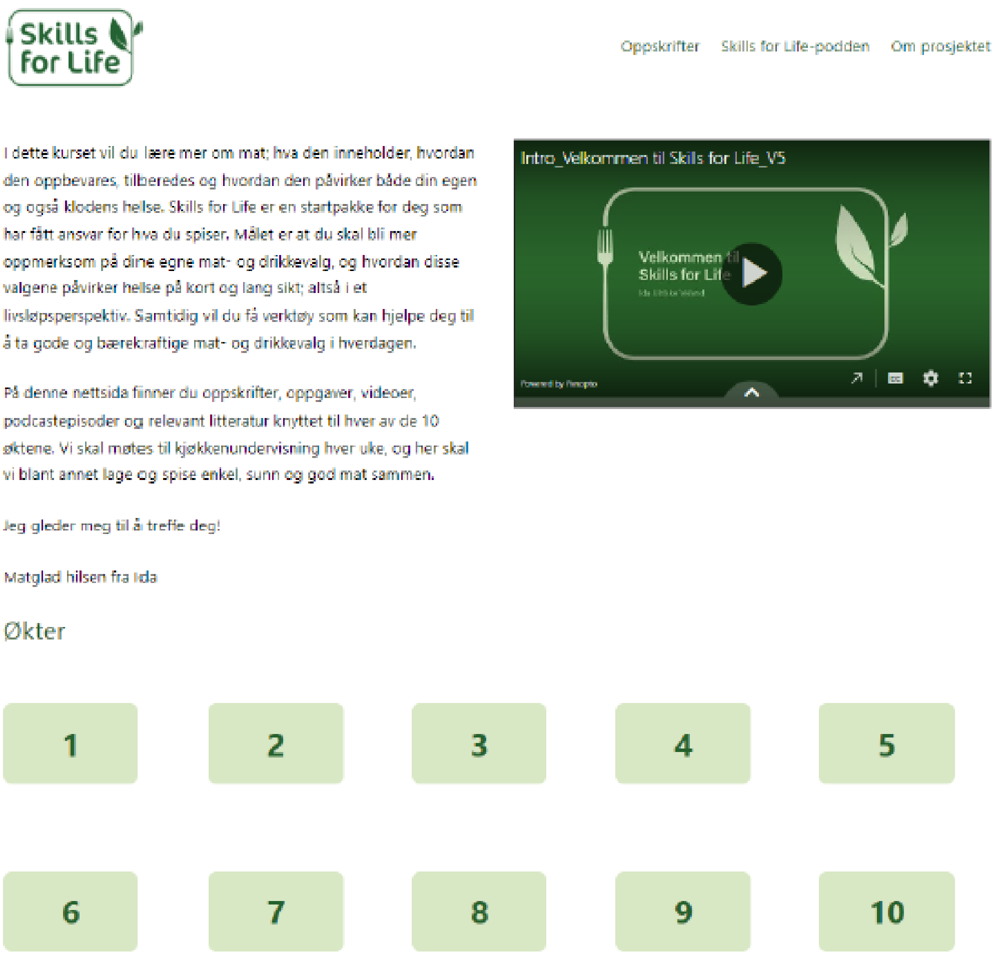

- ☐ Ja, hver dag
- ☐ Ja, 4-6 ganger i uka
- ☐ Ja, 1-3 ganger i uka
- ☐ Ja, 2-3 ganger i måneden
- ☐ Ja, en gang i måneden eller sjeldnere
- ☐ Nei

Har du sett noen av filmene på nettsida? (Introfilm på forsida og én film for hver av de 10 øktene)

- ☐ Ja, 1-3 stk
- ☐ Ja, 4-6 stk
- ☐ Ja, 7-9 stk
- ☐ Ja, 10-11 stk
- ☐ Nei

Har du jobbet med noen av oppgavene på nettsida?

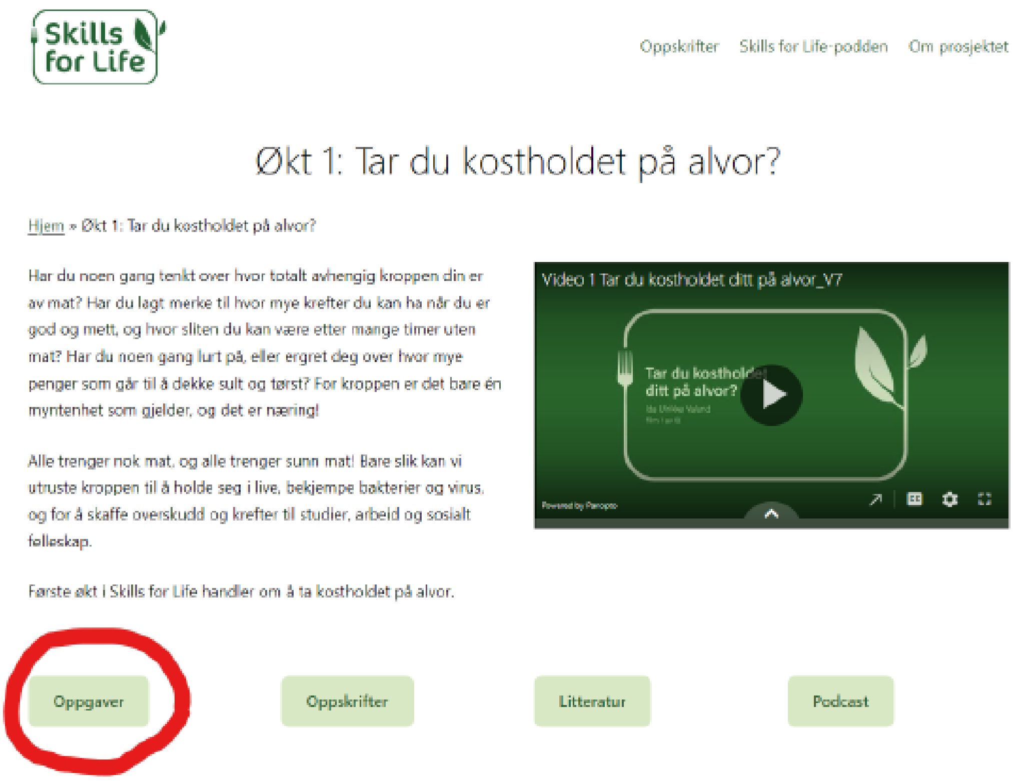

- ☐ Ja, jeg har jobbet med oppgaver for 1-2 økter
- ☐ Ja, jeg har jobbet med oppgaver for 3-5 økter
- ☐ Ja, jeg har jobbet med oppgaver for 6-8 økter
- ☐ Ja, jeg har jobbet med oppgaver for 9-10 økter
- ☐ Nei

Har du sjekket litteraturen for de ulike øktene?

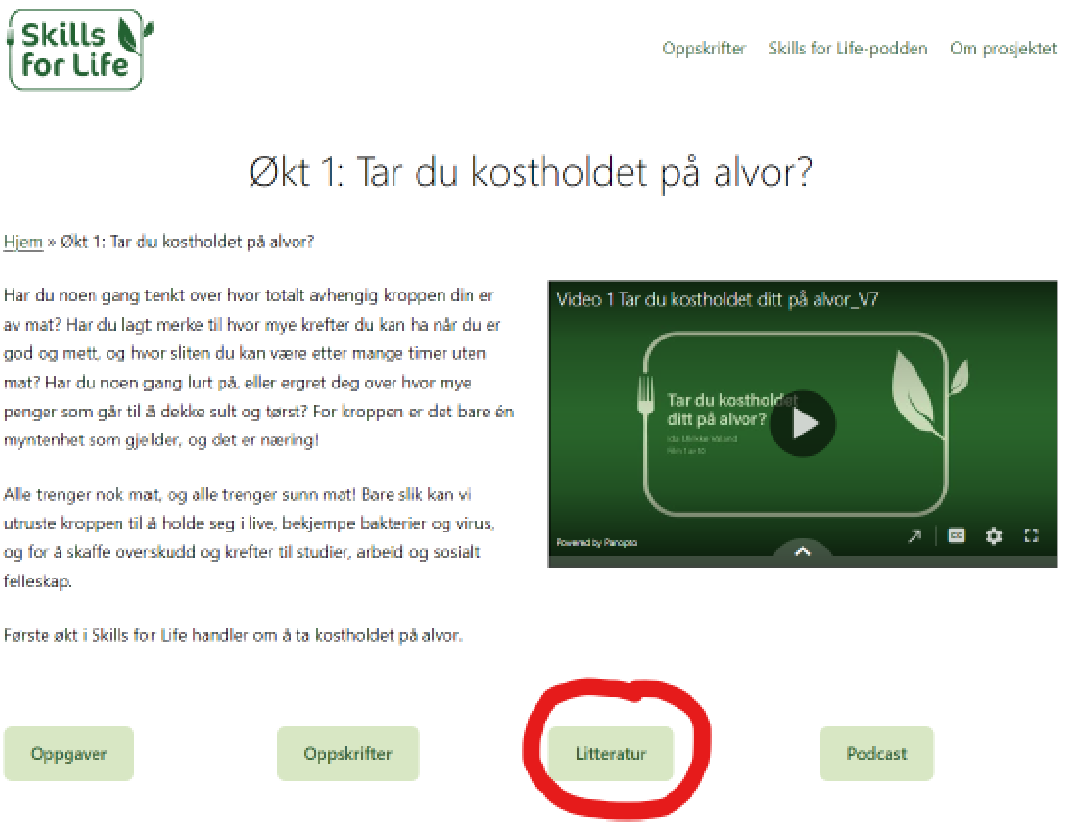

- ☐ Ja, noe
- ☐ Ja, mye
- ☐ Nei

Har du hørt på Skills for Life-podden?

Skills for Life-podden

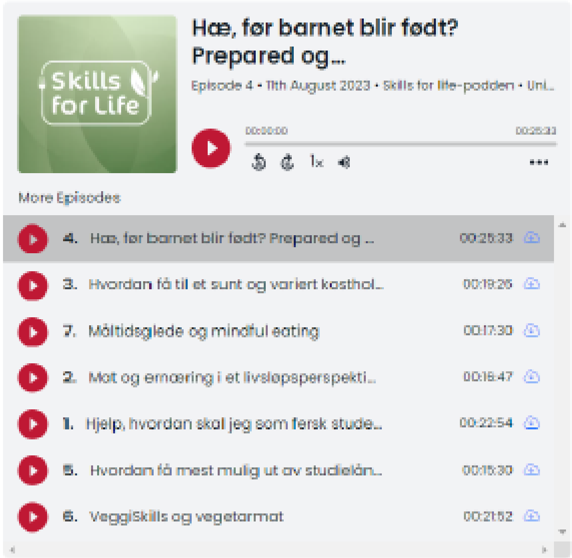

- ☐ Ja, 1-2 episoder
- ☐ Ja, 3-5 episoder
- ☐ Ja, 6-7 episoder
- ☐ Nei

Har du utfyllende kommentarer eller endringsforslag?

Questionnaire 12 – English translation

This is a survey in which we seek your honest feedback on **the entire Skills for Life course**, including all 10 practical kitchen sessions and the accompanying content on the Skills for Life website (including videos, assignments, recipes, literature suggestions, and the Skills for Life podcast).

The survey takes approximately 5-10 minutes to complete.

Thank you very much for your feedback!

How did you find out about the course?

- ☐ I saw an advertisement on Facebook
- ☐ I saw the promotional video on one of the screens at UiA
- ☐ I saw/got a flyer
- ☐ I saw the roll-up in the entrance hall
- ☐ I noticed the stand during the opening ceremony
- ☐ My lecturer informed us about the course during a lecture
- ☐ I heard about it from a friend/acquaintance
- ☐ I heard about Skills for Life at the Be a Student! course organised by UiA PULS at the start of the academic year.
- ☐ Other, please specify

What made you sign up for Skills for Life?

How many of the 10 kitchen sessions have you attended? If you don't remember, please indicate an approximate number.

- ☐ 0
- ☐ 1
- ☐ 2
- ☐ 3
- ☐ 4
- ☐ 5
- ☐ 6
- ☐ 7
- ☐ 8
- ☐ 9
- ☐ 10

What motivated you to attend the course sessions?

What, if anything, prevented you from attending the sessions?

We would like you to evaluate the statements below by ticking the face you think fits best:

The topic of the Skills for Life course is important for me to learn about

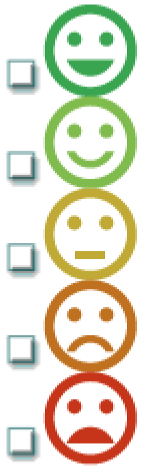

I liked the Skills for Life course

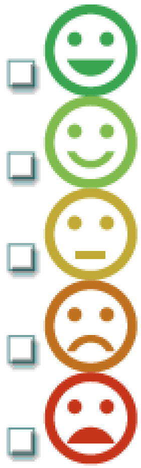

I learned something new from the Skills for Life course

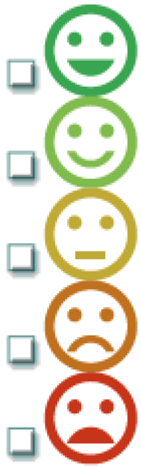

Other students would like the Skills for Life course

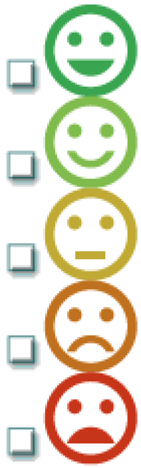

It is important for students to attend the Skills for Life course

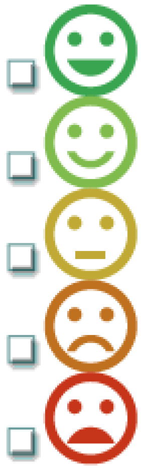

The group work, activities and cooking in the practical cooking sessions were appropriate, educational, and useful

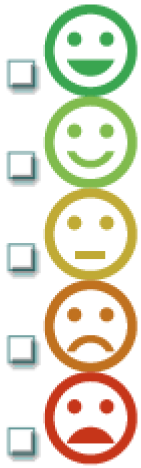

Have you used any of the recipes from Skills for Life at home (either recipes from the practical sessions or from the Skills for Life website)?

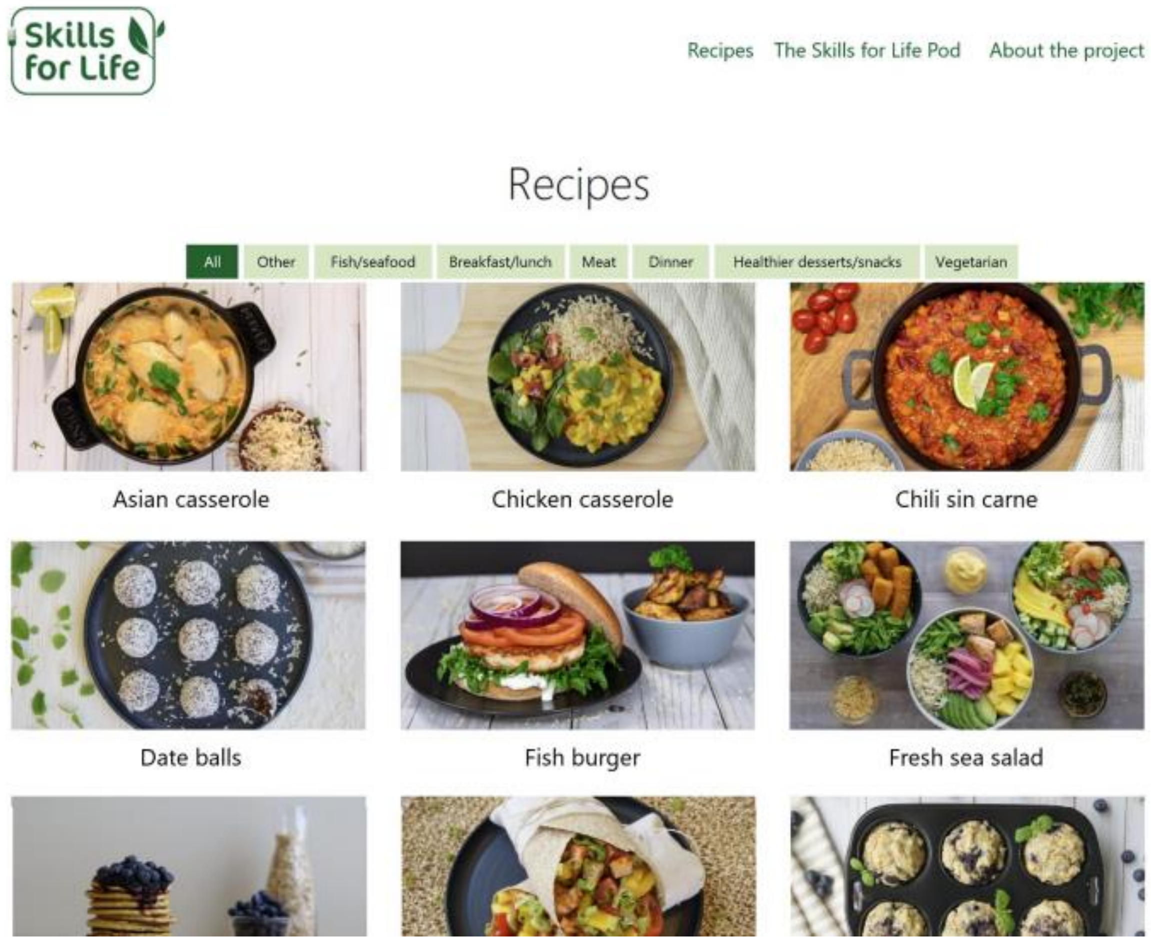

- [ ] Yes, many
- [ ] Yes, a few
- [ ] Don't know
- [ ] No

If you have used any of the recipes at home, which ones?

Has the course affected your daily life?

- [ ] Yes, a lot
- [ ] Yes, a little
- [ ] Don’t know
- [ ] No

If the course has impacted your daily life, how?

Has the course affected how you are feeling?

- [ ] Yes, a lot
- [ ] Yes, a little
- [ ] Don’t know
- [ ] No

If the course has affected your well-being, describe the change:

Have you used the Skills for Life website?

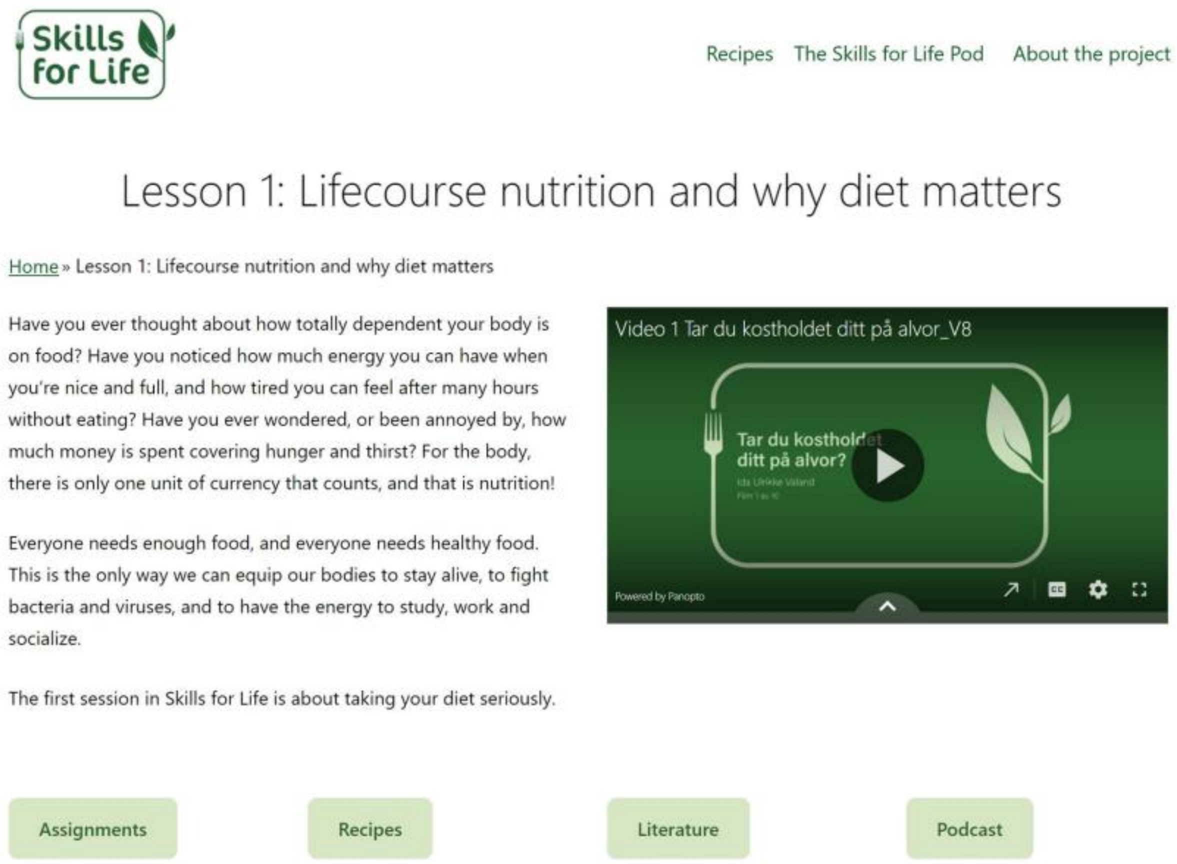

- [ ] Yes, every day
- [ ] Yes, 4-6 times a week
- [ ] Yes, 1-3 times a week
- [ ] Yes, 2-3 times a month
- [ ] Yes, once a month or less
- [ ] No

Have you watched any of the videos on the website? (Introductory video on the homepage and one video for each of the 10 sessions)

- [ ] Yes, 1-3 videos
- [ ] Yes, 4-6 videos
- [ ] Yes, 7-9 videos
- [ ] Yes, 10-11 videos
- [ ] No

Have you worked on any of the assignments on the website?

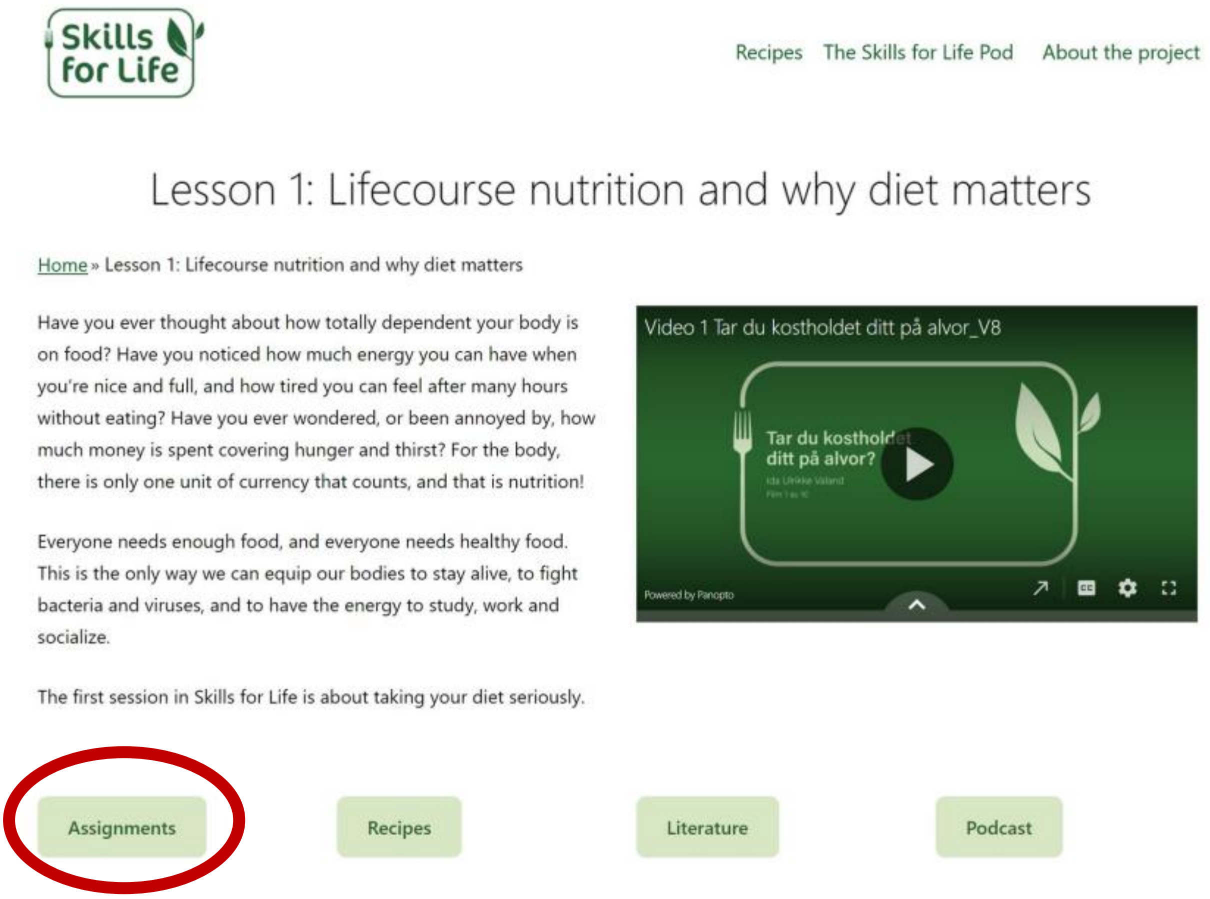

- ☐ Yes, I have worked on assignments for 1-2 sessions.
- ☐ Yes, I have worked on assignments for 3-5 sessions.
- ☐ Yes, I have worked on assignments for 6-8 sessions.
- ☐ Yes, I have worked on assignments for 9-10 sessions.
- ☐ No

Have you looked into the literature for the different sessions?

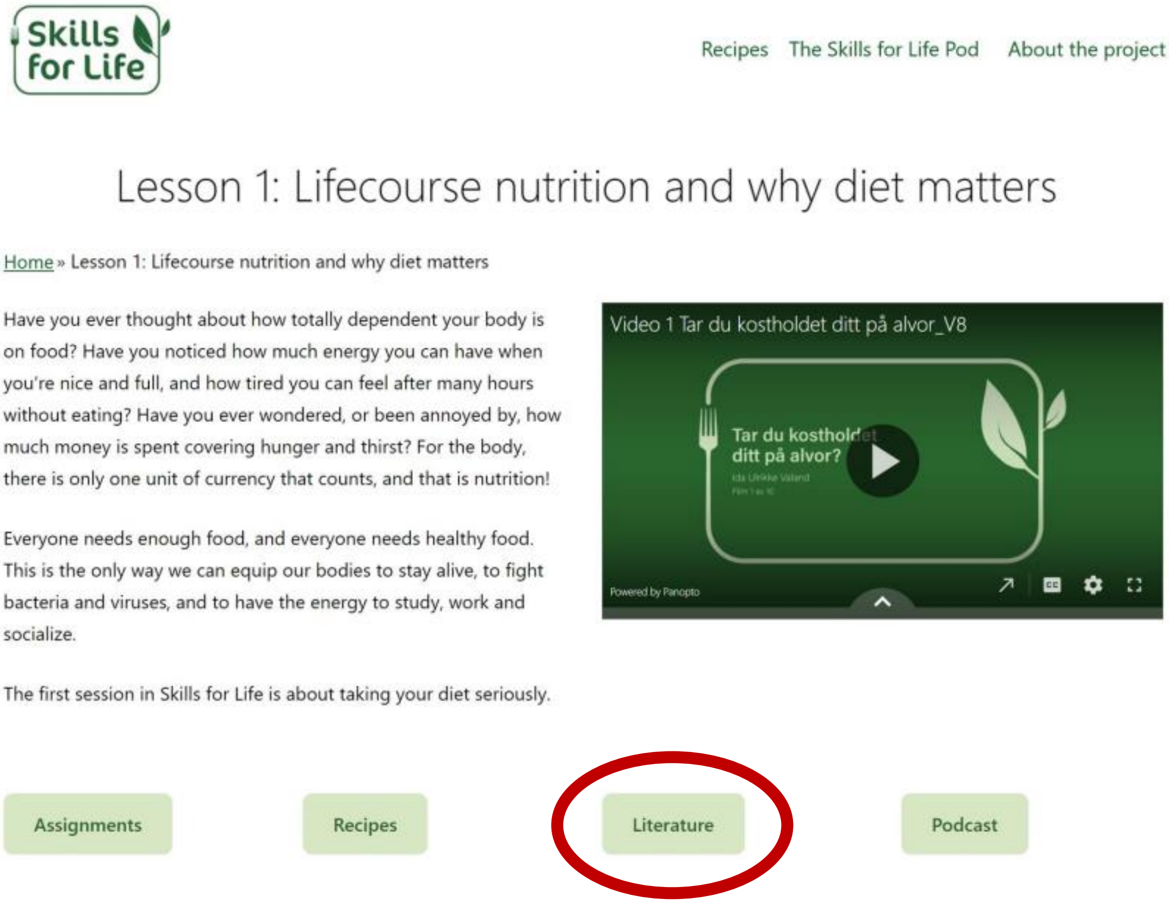

- [ ] Yes, some
- [ ] Yes, a lot
- [ ] No

Have you listened to the Skills for Life podcast?

Skills for Life-podden

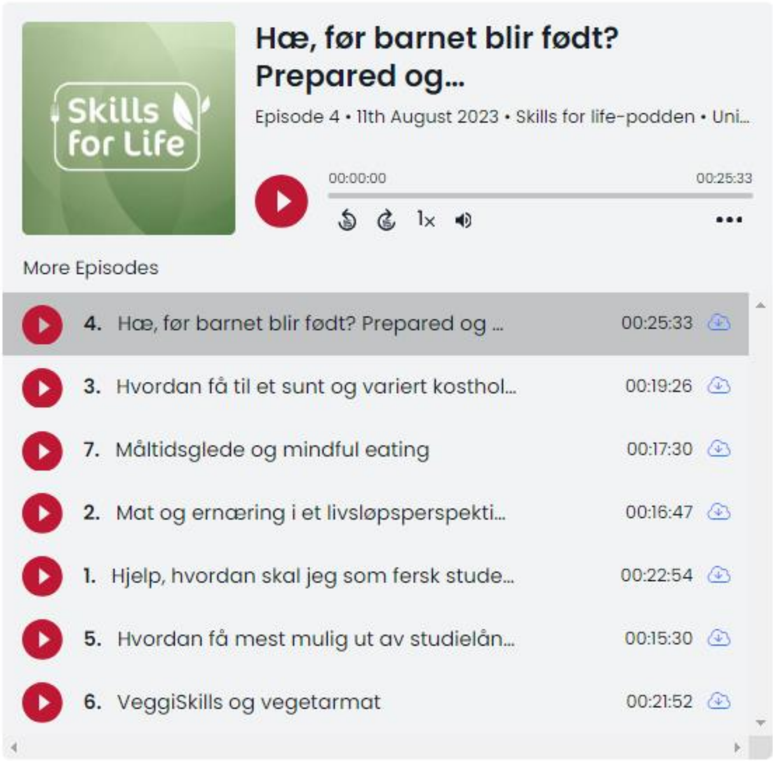

☐ Yes, 1-2 episodes
☐ Yes,3-5 episodes
☐ Yes, 6-7 episodes
☐ No

Do you have any additional comments or suggestions for changes?

