# Supplementary material for: Feasibility of a dietary life skills course aimed at fostering cooking skills and a healthy diet among university students
Source: Pilot Feasibility Stud. 2025 Jul 17;11:100. doi: 10.1186/s40814-025-01680-y (PMC12273360; doi:10.1186/s40814-025-01680-y)
Supplement: Supplementary file 3 — Additional file 3. Overview of learning objectives and the 10 Skills for Life cooking lessons. [file 40814_2025_1680_MOESM3_ESM.pdf]

Additional file 3: Overview of learning objectives and the 10 *Skills for Life* cooking lessons

Skills for Life comprised 10 practical cooking lessons held at the university teaching kitchen, complemented by a website featuring short video lectures, selected literature and learning activities for each lesson. The overarching learning objectives of the course were grounded in the 11 food literacy components suggested by Vidgen and Gallegos[1] (objective 1-11 below). Emphasis was placed on the first four and the 12th objectives.

1. **Make a good tasting meal from whatever food is available (with or without a recipe)**
2. **Apply basic principles of safe hygiene and handling**
3. **Understand food has an impact on personal health and well-being**
4. **Join in and eat in a social way**
5. Prioritise money and time for food
6. Plan food intake
7. Make feasible food decisions
8. Access food through multiple sources and know the advantages and disadvantages of these
9. Determine what is in a food product, how to store and use it
10. Judge the quality of food
11. Demonstrate self-awareness of the need to personally balance food intake
12. **Plan and prepare quick, easy, sustainable, affordable, healthy and tasty dishes**

Specific learning objectives for each of the 10 lessons are presented below.

Week 35: Nutrition from a life course perspective and why diet matters

Learning objectives:

- To understand the relationship between diet and health in a life course perspective
- To cook a simple and balanced meal from scratch, using a recipe

Group assignment: Blind taste tests with orange juice (fresh vs. concentrate), chocolate milk (with vs. without added sugar), ham (regular vs. budget brand), smoothie (bought vs. homemade) and bread (much vs. less wholegrain).

Cooking: Omelette, chicken wok (fresh vs. frozen vegetables) and tomato soup (homemade vs. instant).

Week 36: How to eat healthily

Learning objectives:

- To recognise the Norwegian dietary guidelines

[1] Vidgen HA, Gallegos D. Defining food literacy and its components. Appetite. 2014;76:50-9.

- To apply the plate model as a tool for balancing meals
- To create a balanced food plan by applying the dietary guidelines and the plate model
- To cook a breakfast/lunch alternative, using a recipe

Assignment: Create a meal plan using the plate model and dietary guidelines.

Cooking: Oatmeal pancakes, chia jam, oatmeal porridge, pasta salad and savoury muffins.

Week 38: How to store food to avoid food waste

Learning objectives:

- To understand what food safety is and why it is important
- To practice good kitchen hygiene to avoid cross contamination
- To employ basic principles regarding food storage to avoid food waste
- To employ basic principles to judge the quality of food
- To understand the difference regarding "best before" and "expiry date"
- To cook a simple and balanced dinner, using a recipe

Demonstration: How to cut onions and garlic. Hygiene experiment with onions and melon (cross-contamination). Best before vs. use by dates. Look - smell - taste.

Cooking: Bali chicken casserole, soy salmon, oven baked cod, fish sticks and oven baked apples.

Week 39: How to stock your kitchen

Learning objectives:

- To list basic food items and equipment useful to stock your kitchen
- To cook a quick, healthy and sustainable meal from staple food available at the kitchen by applying the plate model and dietary guidelines

Group assignment: Healthify a dinner meal (pancakes with jam and lemonade) according to the plate model and dietary guidelines.

Cooking: Make a quick, healthy and sustainable dinner from staple foods.

Week 41: Preconception diet

Learning objectives:

- To understand the importance of preconception diet for one's own and potential children's health
- To apply the plate model and dietary guidelines to healthify (modify) a recipe by analysing where there are room for improvements
- To prepare a soup or stew by adapting a basic recipe

Group assignment: Healthify a pizza recipe according to the plate model and dietary guidelines.

Cooking: Various soups or stews following a basic recipe.

Week 42: Food labels and how to interpret them

Learning objectives:

- To understand how food labels are read
- To compare different food items regarding content of specific nutrients
- To make slow-risen bread and homemade spreads as affordable, nutritious and sustainable breakfast or lunch alternatives, using recipes

Group assignment: Rank packaging by content of salt, sugar, saturated fat and fiber.

Cooking: Homemade bread and spreads.

Week 43: How to make the most of your student loan

Learning objectives:

- To apply basic principles to save money on food
- To plan and cook a balanced dinner for less than NOK 120

Group assignment and cooking: Plan and cook dinner for 4 for less than NOK 120.

Week 44: Sustainable food and "food rescuing"

Learning objectives:

- To understand that food has a great impact on the environment
- To identify and compare the water usage required to produce various food items
- To apply basic principles regarding sustainable diet such as reducing meat intake and avoiding food waste
- To cook vegetarian food dishes with a recipe

Assignment: Rank foods by water consumption and measure water needed to produce cheese, beef, pork, eggs, legumes and root vegetables.

Cooking: Vegetarian food (mushroom burger, baked fiesta cauliflower and barleyotto).

Week 45: What is true about food and health?

Learning objectives:

- To be aware that not everything one hear concerning food and health is true
- To critically judge dietary information
- To cook Indian dishes and healthier desserts, using recipes

Discussion: Myth-busting.

Cooking: Indian food (butter chicken and daal), healthier desserts and homemade plant milk.

Week 46: Cooking competition and mindful eating

Learning objectives:

- To set the table for a festive dinner
- To identify the potential benefits of mindful eating and know how to apply this
- To cook a tapas dish of one’s own choice for a festive dinner

Demonstration: How to set a table. Brief introduction on mindful eating.

Cooking: The students chose their own tapas dishes and we enjoyed a festive meal together.
