# Supplementary material for: Feasibility of a dietary life skills course aimed at fostering cooking skills and a healthy diet among university students
Source: Pilot Feasibility Stud. 2025 Jul 17;11:100. doi: 10.1186/s40814-025-01680-y (PMC12273360; doi:10.1186/s40814-025-01680-y)
Supplement: Supplementary file 4 — Additional file 4. ’MyFoodMonth 1.1’ Diet Quality Score scorings. [file 40814_2025_1680_MOESM4_ESM.pdf]

Additional file 4: 'MyFoodMonth 1.1' Diet Quality Score scorings

**Diet Quality Score components**<br>Screener variable(s)	**Scoring Valence**	**Criteria for min score (0)**	**Criteria for max score (10)**	**'MyFoodMonth 1.1' Diet Quality Score scorings**							
				**Never**	**1 a week**	**2-4 a week**	**5-6 a week**	**1 a day**	**2-3 a day**	**4-5 a day**	**≥6 a day**
**Vegetables**<br>Vegetables, including salad, cabbage, carrot, green beans, etc. (not potatoes or sweet potatoes)	positive	≤1 x month	≥4 x day	0	2	4	6	8	9	10	10
**Fruits**<br>Fruit and berries, including fresh, frozen, and canned (not juice or smoothie)	positive	≤1 x month	≥2 x day	0	2	4	6	8	10	10	10
**Whole grain (products)**<br>Cereal and porridge, Unsweetened (e.g., 4-Korn muesli, oatmeal, Go'dag muesli, and Weetabix)<br>Whole grain bread, crispbread, rolls (>50% whole grain)<br>Whole grain dinner products (e.g., barley, pasta, couscous)	positive	≤1 x month	2-5 x day	0	2	4	6	8	10	10	8
**Sugar-sweetened beverages**<br>Sugar-sweetened beverages<br>Sugar-sweetened energy drinks (e.g., Gatorade, Red Bull)	negative	≥1 x day	0	10	6	4	1	0	0	0	0
**Sugary foods**<br>Cereal and porridge, Sweetened (e.g., Special K, Corn Flakes with honey)<br>Candy, including chocolate<br>Waffles, buns, cake, biscuits etc.<br>Ice cream, panna cotta, pudding, mousse, etc.	negative	≥1 x day	0	10	6	4	1	0	0	0	0
**Beans and lentils**<br>Beans, lentils, chickpeas, peas (not green beans)	positive	0	≥2 x day	0	4	6	8	9	10	10	10
**Nuts and seeds (unsalted)**<br>Unsalted nuts and seeds	positive	0	1-3 x day	0	4	6	8	10	10	8	6
**Meat (processed and red)**<br>Red meat, minced or cuts (beef, lamb, pork, goat)<br>Processed meat (e.g., bacon, spread, sausage)	negative	≥2 x day	≤1 x month	10	6	4	2	1	0	0	0
**Fish**<br>Fatty fish and fish products (e.g., salmon, mackerel)<br>Lean fish and fish products (e.g., cod, pollock)<br>Fish spread (e.g., mackerel in tomato sauce)	positive	0	2 x week	0	8.5	10	10	10	10	10	10
**Salty foods**<br>Salty snacks (e.g., popcorn, chips, salty nuts)	negative	≥2 x day	0	10	6	4	2	1	0	0	0

Total Possible Points: 100

Modified version of the scoring system by Salvesen et al.

Salvesen L, Wills AK, Øverby NC, Engeset D, Medin AC. Relative validity of a non-quantitative 33-item dietary screener with a semi-quantitative food frequency questionnaire among young adults. Journal of Nutritional Science. 2023;12:e72.
